# High-value valorization of *Periplaneta americana* residue: intestinal health regulation in pre-bred hens and circular economy applications

**DOI:** 10.3389/fvets.2025.1700996

**Published:** 2025-12-11

**Authors:** Sicai Tao, Shunyi Zi, Liangjing Yan, Mei Yang, Tianzhang Zhao, Huiying Li

**Affiliations:** 1 College of Agriculture and Biological Sciences, Dali University, Dali, China; 2 Yunnan Provincial Key Laboratory of Entomological Biopharmaceutical R&D, College of Pharmacy, Dali University, Dali, China; 3 National-Local Joint Engineering Research Center of Entomoceutics, Dali University, Dali, China

**Keywords:** *Periplaneta americana* residue, growth performance, intestinal health, intestinal microbiota, transcriptomic

## Abstract

This study investigated the effects of *Periplaneta americana* residue (PAR) on the growth performance and gut health of laying hens during the pre-breeding phase. Over an 11-week period, 450 one-month-old Nandan Yao chickens were assigned to six dietary groups in which PAR replaced 0%, 10%, 20%, 30%, 40%, or 50% of the soybean meal. The results revealed no significant impact of PAR on growth performance (*p* > 0.05). However, PAR supplementation significantly elevated duodenal and jejunal immunoglobulin A (IgA) levels, as well as ileal immunoglobulin M (IgM) and immunoglobulin G (IgG) levels (*p* < 0.05). Although no overall significant differences were observed in the villus height (VH), width (VW), crypt depth (CD), or the villus-to-crypt (V/C) (*p* > 0.05), ileal VH and V/C increased specifically in certain groups (*p* < 0.05). Additionally, ileal and jejunal pH values decreased significantly with increasing PAR levels (*p* < 0.05), and the intestinal microbiota diversity remained stable. Transcriptomic analysis revealed the regulatory roles of PAR in RNA biosynthesis, DNA repair, the immune response, and metabolic pathways. Overall, PAR incorporation modestly enhanced the growth performance and intestinal health in laying hens. These findings support PAR as an effective substitute for soybean meal in poultry diets, with a replacement ratio of 30% to 50%, growth performance, demonstrating optimal efficacy. This approach not only alleviates pressure on protein resources but also provides a scientific framework for valorizing insect-derived waste within circular agriculture systems.

## 1 Introduction

In the global food security landscape, the protein supply is grappling with the twin imperatives of unyielding limitations on arable land and escalating ecological burdens. The increasing expenditures and intrinsic ecological vulnerabilities of traditional protein sources have constituted a significant impediment to industrial progress, thereby compelling the pursuit of transformative alternative approaches (1). Insect proteins, distinguished by their remarkable biological conversion efficiency and negligible ecological impact, have the potential to become pivotal strategic resources, heralding a profound “protein revolution” (2). Against this backdrop, the consecutive enactment of legislative frameworks such as EU Regulation (EU) 2017/893 and China’s “Three-Year Action Plan for Reducing and Replacing Soybean Meal in Feed” has imparted substantial momentum to the industrial deployment of insect protein, thereby underscoring the elevation of sustainable protein resource development to a national strategic imperative (3, 4).

The *Periplaneta americana* (PA) stands as a pre-eminent species within the Blattidae family, distinguished by its considerable scientific and economic salience. Its utility exceeds that of many conventional insect species; it is not only a nutritional paragon, replete with superior proteins, meticulously balanced amino acids, and efficacious functional lipids but also a veritable fount of natural bioactive compounds. Its immunomodulatory and antimicrobial attributes have been comprehensively substantiated in the pharmaceutical field (5, 6). Well-established pharmaceuticals such as Kangfuxin Liquid and Xinmailong Injection serve as illustrative testaments for their profound therapeutic efficacy (7, 8). However, this remarkable medicinal utility is juxtaposed with the generation of considerable pharmaceutical residue after extraction—a largely unappreciated resource indicative of a significant “value disparity.” Traditional disposal methods, such as incineration, composting, or biodiesel production, not only cause secondary ecological contamination but also result in substantial squandering of valuable nutrients and bioactive constituents (9). Investigations revealed that the crude protein content of PAR is as high as 73.41%, that of crude fat is 20.48%, that of crude fiber is 10.25%, and that of crude ash is 6.36% (on a dry matter basis). Furthermore, the natural antimicrobial peptides and other active substances present in PAR present novel avenues for inquiry into poultry protein and antibacterial feed. This approach not only helps mitigate the dearth of protein resources and curtail husbandry expenditures but also confers supplementary ecological advantages. Studies have revealed that integrating PAR into feed has no deleterious effect on the growth trajectory of juvenile Nile tilapia and, to a certain degree, augments murine immunity (10, 11). Nevertheless, the extant body of research concerning the practical implications and underlying mechanisms of PAR in laying hens, especially within their pivotal developmental phase, remains conspicuously nascent, highlighting its promise as an innovative protein source for feed, potentially rivaling or even eclipsing the economic value of soybean meal (approximately 3 RMB/kg).

This study is predicated upon this pivotal “waste-to-resource” metamorphosis, employing the esteemed indigenous Chinese breed, the Nandan Yao chicken, as the experimental cohort. It specifically addresses the nascent rearing phase, recognized as a crucial “nutritional aperture” fundamentally dictating lifelong productivity. Intestinal development and health during this stage are integral foundations that underpin subsequent oviparous capacity and immunological resilience (12, 13). To bridge the lacuna in current research concerning the deployment of PAR in laying hen husbandry, this study employs the Nandan Yao chicken as a representative model. By integrating graded concentrations of PAR into the feed regimen as a substitute for extruded soybean meal, we comprehensively investigated its multifaceted effects on the growth metrics, intestinal architecture, immunoglobulin profiles, luminal pH, microbial community composition, and transcriptome dynamics of laying hens during their early rearing period. This research transcends the scientific corroboration of an alternative feed strategy to embody an innovative practical application of a circular economic paradigm. It endeavors to forge an integrated industrial continuum spanning “pharmaceutical byproduct valorization—premium feed formulation—sustainable animal husbandry,” thereby realizing the holistic valorization of PA resources. The research findings provide substantive theoretical underpinnings and quantifiable directives for the judicious deployment of PAR within the avian husbandry sector, pave the way for novel strategies for the targeted modulation of avian gut health, and, in summary, furnish indispensable scientific acumen and a pragmatic framework for fostering the ecological, efficacious, and enduring transformation and enhancement of China’s livestock sector.

## 2 Materials and methods

### 2.1 Experimental diets

PAR was obtained from Yunnan Teng Pharmaceutical Co. Mature PA samples were collected, subsequently desiccated at temperatures between 80 and 90 °C, and then pulverized to achieve a particle size capable of passing through a 250-μm sieve. This PA was then mixed with 95% ethanol at a mass-to-volume ratio of 1:3, allowed to macerate for five days with bimodal daily agitation, and thereafter subjected to filtration. This extraction procedure was iterated twice, after which the PAR was isolated and air-dried at ambient temperature for subsequent application. The remaining dietary constituents were meticulously blended in accordance with the prescribed feed formulation. Six iso-nitrogenous and iso-lipidic diets were meticulously formulated by substituting 0%, 10%, 20%, 30%, 40%, and 50% of the expanded soybean meal in the foundational diet with PAR (designated diets A, B, C, D, E, and F, respectively; refer to Table 1). Ultimately, the compounded feeds were hermetically sealed within specialized packaging engineered for superior moisture resistance, breathability, compressive integrity, and sealing efficacy. These feeds were subsequently warehoused in a desiccated, well-ventilated facility shielded from direct solar exposure at ambient temperatures between 15 and 25 °C. The fundamental nutritional profiles, heavy metal concentrations, and deleterious microorganism assay outcomes for the PA product are delineated in Table 2.

**Table 1:** Experimental base feeds with nutrient levels and nutrient composition (air-dried base).

Items (%)	A	B	C	D	E	F
Corn	60.48	62.26	63.92	65.98	68.00	69.5
Wheat bran	1.99	2.00	2.05	1.89	1.77	2.00
Expanded soybean meal	24.4	21.96	19.52	17.08	14.64	12.2
Calcium hydrogen phosphate	1.82	1.53	1.25	0.96	0.62	0.32
DL- methionine	0.18	0.17	0.16	0.15	0.14	0.13
Vegetable oil	3.87	3.30	2.76	2.11	1.47	0.98
Premix	5.00	5.00	5.00	5.00	5.00	5.00
Salt	0.41	0.37	0.35	0.28	0.23	0.18
Choline chloride	0.20	0.20	0.20	0.20	0.20	0.20
L-Lysine hydrochloride	0.27	0.26	0.25	0.24	0.23	0.22
Stone powder	1.38	1.30	1.23	1.15	1.10	1.04
PAR	0	1.65	3.31	4.96	6.60	8.23
Total	100.0	100.0	100.0	100.0	100.0	100.0
Nutritional composition						
Metabolizable energy (MJ/kg)	12.01	12.01	12.01	12.01	12.01	12.01
Crude protein (%)	16.0	16.0	16.01	16.01	16.0	16.0
Calcium (%)	1.00	1.00	1.00	1.00	1.00	1.00
βTotal phosphorus (%)	0.63	0.63	0.62	0.62	0.60	0.60
Nonphytate phosphorus (%)	0.38	0.38	0.39	0.39	0.38	0.38
Methionine (%)	0.70	0.70	0.70	0.70	0.70	0.70
Lysine (%)	1.00	1.00	1.00	1.00	1.00	1.00
Methionine (%)	0.43	0.43	0.43	0.44	0.44	0.44

The premix provides for each kilogram of ration: VA 8,000 IU, VD3 3,000 IU, VE 16 IU, VK3 2 mg, VB2 8.3 mg, VB6 2.6 mg, VB12 0.015 mg, niacin 43 mg, folic acid 0.92 mg, calcium pantothenate 12 mg, biotin 0.12 mg, copper 8 mg, iron 8.0 mg, zinc 8.0 mg, manganese 60 mg, iodine 0 35 mg, and selenium 0 15 mg. mg, zinc 8 0 mg, manganese 6 0 mg, iodine 0.35 mg, and selenium 0.15 mg. The metabolizable energy is calculated, and the others are measured values.

**Table 2:** The basic nutritional components, heavy metal contents, and harmful microorganism test results of the PAR.

Items	Test results	Safety range
Crude protein content	73.41%	
Crude fat	20.48%	
Crude fiber	10.25%	
Crude ash	6.36%	
Mercury (mg/kg)	0.0712	≤10
Arsenic (mg/kg)	Not detected	≤2
Cadmium (mg/kg)	Not detected	≤0.5
Chromium (mg/kg)	Not detected	≤2
Mucedine (CFU/g)	<10	<20,000
Salmonella (CFU/25 g)	Not detected	

### 2.2 Experimental setup and culture management

The experimental investigations were conducted in Dali Bai Autonomous Prefecture, Yunnan Province, China, specifically within the Dianzhong Industrial Park, Dacang Town, Weishan Yi and Hui Autonomous Counties. For this study, a cohort of 450 one-month-old Nandan Yao chickens, which presented comparable body weights, was procured from a local commercial farm, where they had undergone comprehensive vaccination according to standard immunization protocols. These 450 Nandan Yao chickens were subsequently allocated at random into six distinct treatment groups, each comprising five replicates of 15 chickens. Analysis of variance (ANOVA) confirmed the absence of significant disparities in initial body weights across these groups. The experimental phase extended over a duration of 11 weeks. Group A, which served as the control, received a foundational diet, whereas groups B, C, D, E, and F were administered experimental diets in which 10%, 20%, 30%, 40%, and 50% of the expanded soybean meal, respectively, had been supplanted by PAR. During the seven-day acclimatization period preceding the commencement of the main experimental phase, six chickens initially displayed suboptimal health; however, their condition normalized after isolation.

Throughout the experimental duration, cooperative management diligently upheld rigorous protocols for disinfection, ventilation, and overall sanitation. Photoperiod management was meticulously calibrated to align with the developmental requirements of the laying hens, commencing with 23 h of illumination during the initial week and progressively decreasing by 1 hour weekly until a consistent photoperiod of 16 h was attained. The ambient indoor temperature was maintained at 32 °C during the initial week and subsequently gradually decreased to 2 to 3 °C per week until it stabilized at 24 °C. The relative humidity was precisely regulated within a range of 50% to 60%, and ad libitum access to feed and potable water was provided to the chickens throughout the feeding period.

### 2.3 Measurement of growth performance

Upon formal commencement of the experiment, all Nandan Yao chickens were subjected to a fasting period before their initial body weights were meticulously recorded. Throughout the experimental duration, weekly body weight measurements were systematically acquired to track the growth trajectory of each treatment cohort comprehensively. Three pivotal performance metrics were subsequently calculated: the average daily gain (ADG), the average daily feed intake (ADFI), and the feed conversion ratio (F/G). These indices were derived via the following specific formula: ADG was calculated as the difference between the final and initial body weights of the hens divided by the total number of feeding days; ADFI represented the cumulative feed consumption of the hens divided by the duration of the experimental feeding period; and F/G was obtained by dividing the ADFI by ADG.

### 2.4 Sample collection

Prior to the commencement of sampling, each experimental cohort underwent a 12-h fasting period, although ad libitum access to potable water was maintained. During the eleventh week of the experimental phase, one chicken of comparable body weight was selected from each replicate and humanely euthanized. Euthanasia was performed subsequent to intravenous administration of sodium pentobarbital (0.3 mL/kg body weight) to induce anesthesia, followed by exsanguination via a cervical incision. Immediately after euthanasia, approximately 2 grams of luminal contents were aseptically harvested from the duodenum, jejunum, ileum, and cecum through an abdominal dissection. These samples were then transferred into sterile cryogenic vials, rapidly cryopreserved in liquid nitrogen, and subsequently stored at 80 °C for future analytical procedures. Concurrently, the intestinal segments were meticulously irrigated with saline solution and carefully blotted dry using filter paper. Precisely 4 cm in length was excised from the duodenum, jejunum, and ileum. Each excised segment was then bisected: One portion was immediately immersed in 4% paraformaldehyde for fixation and stored at ambient temperature, while the other portion was swiftly transferred into a 2 mL cryovial, snap-frozen in liquid nitrogen, and stored at −80 °C.

### 2.5 Determination of intestinal immunoglobulins and cytokines

Immunoglobulin and cytokine concentrations were precisely quantified via enzyme-linked immunosorbent assay (ELISA). For the initial coating step, the primary antibody was diluted in carbonate coating buffer to achieve protein concentrations ranging from 1 to 10 μg/mL. One hundred microliters of this solution was dispensed into each well of a polystyrene microplate, which was then incubated overnight at 4 °C. The wells subsequently underwent three successive washes, each lasting 3 min, with the designated washing buffer. To prevent nonspecific binding, 200 μL of blocking solution was then added to each well, and the plate was incubated at 37 °C for a period of 12 h. Next, the blocking solution was decanted, and the wells were again subjected to three washes with washing buffer. Next, 100 μL of appropriately diluted samples, alongside blank control wells and serially diluted standards, were added to the respective wells. The plate was then incubated at 37 °C for 12 h, sealed with a plate-sealing film, and subjected to another three washing cycles. Subsequently, 100 μL of a diluted biotinylated antibody working solution was added to each well, followed by incubation at 37 °C for 1 h and an additional washing step. Thereafter, 100 μL of a diluted enzyme conjugate working solution was added, and the plate was incubated for 30 min at 37 °C in the dark before one final wash. A chromogenic substrate was then introduced by dispensing 100 μL of TMB substrate solution into each well, and the plate was incubated at 37 °C and shielded from light for 10 to 30 min until a distinct color gradient became discernible within the standard wells. The enzymatic reaction was terminated by adding 100 μL of 2 M sulfuric acid to each well, resulting in a characteristic color transition from blue to yellow. Finally, within a 10-min window, the optical density (OD) of each well was precisely measured at 450 nm via a microplate reader, with the blank control well serving as the zero reference. A standard curve was meticulously constructed using the known concentrations of the standards and their corresponding OD values, from which the concentrations of the experimental samples were subsequently interpolated.

### 2.6 Determination of intestinal morphology

After dehydration, embedding, and staining of the fixed duodenum, ileum, and jejunum with 4% paraformaldehyde, the standard operating procedure (SOP) for pathological laboratory examination developed by Wuhan Xavier Biotechnology Company was followed. A PANNORAMIC panoramic section scanner was employed to scan the qualified samples and generate a folder containing all the tissue information. Subsequently, CaseViewer 2.4 software was utilized for 20x imaging, and the height and width of five intact villi, as well as the depth of the crypts in each section, were measured precisely using Image-Pro Plus 6.0, with millimeters as the standardized metric. The villus-to-crypt ratio (V/C) was then calculated.

### 2.7 Determination of intestinal pH

The pH values of the contents of the jejunum, duodenum, and cecum were measured directly via a pH B-8 pen pH meter. The electrodes were rinsed after each measurement, and the results were recorded.

### 2.8 Analysis of the intestinal flora

The contents of the cecum were frozen in liquid nitrogen and sent to Shanghai Meiji Biopharmaceutical Technology Co. All the data were analyzed on the Meiji life cloud platform^1^ as follows: Alpha diversity metrics, including the Chao and Shannon indices, were calculated via the appropriate software. The Wilcoxon rank-sum test was employed to analyze the differences in alpha diversity between groups.

### 2.9 Analysis of the intestinal transcriptome

Total RNA was successfully extracted from jejunal tissues via a dedicated RNA extraction kit supplied by Shanghai Meiji Biotechnology Co. The concentration and purity of the isolated RNA were precisely determined spectrophotometrically. Furthermore, the integrity of the RNA was initially verified through agarose gel electrophoresis to confirm the quality of the samples.

Subsequent to these quality evaluations, library construction was carried out by Shanghai Meiji Biotechnology Co. For the raw sequencing data, rigorous quality control was implemented via fastp software to effectively remove impurities and low-quality sequences that could compromise downstream analysis. The quality-controlled sequences were then accurately aligned to the *Gallus gallus* reference genome using HISAT2, thereby yielding crucial transcript information for subsequent transcriptome assembly and quantitative expression analysis.

To precisely quantify the expression levels of genes and transcripts, RSEM software, which is known for its efficiency in calculating and analyzing gene/transcript expression differences across diverse samples, was employed. Differential expression analysis was conducted via DESeq2, applying stringent screening criteria: A differential fold change (|log2FC|) of greater than or equal to 1 and a false discovery rate (FDR) of less than 0.05 (for DESeq2) or less than 0.001 (for DEGseq) to identify significantly differentially expressed genes.

Moreover, the identified DEGs were subjected to Gene Ontology (GO) and Kyoto Encyclopedia of Genes and Genomes (KEGG) enrichment analyses. These analyses, which employed the Benjamini–Hochberg multiple test correction method, aimed to elucidate the potential roles of these genes in various biological processes. GO functional enrichment analyses were performed via the GO database, whereas KEGG pathway analyses were conducted via the scipy package in Python, thereby ensuring the accuracy and robustness of the analyses.

### 2.10 Data processing and analysis

All the data were initially organized via Excel 2019 software and subsequently analyzed via one-way analysis of variance (ANOVA) between groups via SPSS 27.0. All values are expressed as the means ± standard deviations. Differences were significant (*p* < 0.05), and Duncan’s multiple comparisons test was performed. Differences were significant (*p* < 0.05), then Duncan’s multiple comparisons were performed.

## 3 Results

### 3.1 Growth performance

Table 3 details the impact of PAR supplementation on the growth performance of laying hens during their pre-breeding phase. The inclusion of varying proportions of PAR as a substitute for soybean meal in the diet did not induce any statistically significant differences (*p* > 0.05) in the average growth performance parameters among the experimental groups. Nevertheless, the ADG and F/G in Groups B, C, E, and F were greater than those recorded for control Group A.

**Table 3:** Effects of PAR on the growth performance of pre-breeding laying hens.

Items	Groups						*p* value		
	A	B	C	D	E	F	SEM	Linear	Quadratic
ADG/g	19.41	21.00	20.93	18.12	20.58	20.92	0.40	0.685	0.845
ADFI/g	100.27	105.68	110.58	103.03	102.71	105.77	1.82	0.774	0.710
F/G	5.25	5.04	5.28	5.74	4.99	5.09	0.10	0.834	0.604

Data with the same letter or no letter are not significantly different, whereas those with different letters are significantly different (P < 0.05). A: 0% PAR replacing soybean meal; B: 10% PAR replacing soybean meal; C: 20% PAR replacing soybean meal; D: 30% PAR replacing soybean meal; E: 40% PAR replacing soybean meal; F: 50% PAR replacing soybean meal. (The following table is the same as the previous one).

### 3.2 Intestinal immunoglobulins

The effects of PAR incorporation on the intestinal immunoglobulin levels of laying hens during the pre-breeding phase are shown in Table 4. In the duodenum, a statistically significant increase in the IgA concentration was observed in treatment Groups B through E compared with that in control group A (*p* < 0.05). Furthermore, a significant quadratic relationship was identified between duodenal IgA levels and the dietary substitution rate of PAR, indicating a progressive and significant increase in IgA concentrations with increasing replacement proportions. Conversely, the IgG levels in Group A were notably lower than those in Groups C through F (*p* < 0.05). This revealed a significant quadratic increase in the IgG concentration in response to increasing PAR substitution ratios (*p* < 0.05), characterized by a distinct secondary increase in the IgG content (*p* < 0.05). Within the ileum, the IgA content in Group A was significantly lower than that in Group D (*p* < 0.05), and a significant quadratic increase was observed in association with the increasing proportion of PAR replacement (*p* < 0.05). In the jejunum, the IgA concentration in Group E was notably greater than that observed in all the other groups (*p* < 0.05). Moreover, the levels of IgM and IgG in Group B, D, and E were significantly elevated compared with those in Group A (*p* < 0.05), with a significant quadratic increase in IgM levels observed as the proportion of PAR substitution increased (*p* < 0.05).

**Table 4:** Effects of PAR on intestinal immunoglobulins in pre-breeding laying hens.

Items		Groups						*P* value		
		A	B	C	D	E	F	SEM	Linear	Quadratic
Duodeum	IGA(mg/L)	704.72^c^	1091.08^ab^	1204.46^a^	935.26^b^	696.81^c^	729.84^c^	50.34	0.208	0.002
	IGM(mg/L)	2076.59^a^	1279.23b	2230.20^a^	1926.29^a^	986.27^b^	1228.53^b^	118.22	0.020	0.044
	IGG (g/L)	12.99^c^	11.76^c^	23.15^a^	23.84^a^	17.82^b^	16.31^bc^	1.18	0.147	0.002
Ileum	IGA (mg/L)	791.99^bc^	638.80^cd^	898.72^b^	1306.80^a^	785.08^bc^	510.73^d^	62.13	0.674	0.014
	IGM(mg/L)	1946.67^a^	1916.61^ab^	1168.92^d^	2226.64^a^	1582.13^bc^	1248.66^cd^	96.27	0.081	0.199
	IGG (g/L)	14.74^a^	14.47^a^	12.41^ab^	9.55^b^	10.90^ab^	10.15^b^	0.57	<0.001	<0.001
Jejunum	IGA (mg/L)	1050.52^bc^	1156.75^ab^	835.77^c^	851.63^c^	1262.35^a^	913.03^c^	40.88	0.670	0.766
	IGM(mg/L)	1025.50^c^	1836.62^b^	1192.61^c^	2041.95^ab^	2306.89^a^	1282.03^c^	120.90	0.159	0.033
	IGG (g/L)	13.85^b^	21.76^a^	13.01^b^	20.02^a^	19.27^a^	15.24^b^	0.86	0.727	0.359

### 3.3 Intestinal cytokines

Table 5 shows the effects of PAR on intestinal cytokine levels in pre-breeding laying hens. In the duodenum, the concentrations of tumor necrosis factor-*α* TNF-(*α*) and interleukin-2 (IL-2) were significantly greater in the specific treatment group (unidentified in the provided text) than in Group A (*p* < 0.05). Furthermore, interferon-*γ* (INF-γ) levels were significantly elevated in Groups C, D, and F relative to those in Group A (*p* < 0.05), demonstrating a significant quadratic relationship with increasing levels of PAR substitution (*p* < 0.05). In the ileum, the IL-2 levels in Group E were significantly greater than those in Group A (*p* < 0.05). Within the jejunum, TNF-α levels were significantly greater in Groups B, C, D, and F than in Group A (*p* < 0.05), exhibiting a notable quadratic increase corresponding to higher levels of PAR substitution (*p* < 0.05). Additionally, INF-γ levels were significantly higher in Goup E than in all other Groups (*p* < 0.05).

**Table 5:** Effects of PAR on intestinal cytokines in pre-breeding laying hens.

Items		Groups						*P* value		
		A	B	C	D	E	F	SEM	Linear	Quadratic
Duodenum	Tnf-α(ng/L)	49.32^b^	46.78^b^	40.97^b^	42.11^b^	41.11^b^	73.15^a^	2.98	0.091	< 0.001
	INF-γ(ng/L)	48.85^c^	65.16^bc^	89.52^a^	89.58^a^	58.42^bc^	70.15^b^	3.96	0.301	< 0.005
	1 L-2(ng/L)	139.42^b^	177.54^b^	134.53^b^	131.61^b^	156.62^b^	292.2^a^	14.16	0.011	< 0.001
Ileum	Tnf-α(ng/L)	89.07^a^	76.87^ab^	42.60^c^	70.42^b^	65.22^b^	71.88^b^	3.57	0.213	< 0.005
	INF-γ(ng/L)	67.19^a^	43.58^bc^	72.93^a^	44.58^bc^	48.78^b^	35.94^c^	3.33	0.008	0.027
	1 L-2(ng/L)	151.45^bc^	185.63^bc^	176.97^bc^	115.06^c^	316.04^a^	201.22^b^	16.05	0.078	0.214
Jeunum	Tnf-α(ng/L)	37.37^c^	69.70^b^	58.12^b^	62.10^b^	44.24^c^	88.85^a^	4.16	0.020	0.065
	INF-γ(ng/L)	66.94^b^	45.54^c^	71.39^ab^	46.98^c^	81.38^a^	62.80^b^	3.29	0.370	0.554
	1 L-2(ng/L)	262.02^ab^	267.43^ab^	290.47^ab^	279.13^ab^	248.07^b^	304.50^a^	5.75	0.236	0.485

These findings collectively suggest that the substitution of soybean meal with PAR enhances intestinal cytokine levels in pre-breeding laying hens, with Groups E and F demonstrating the most pronounced effects.

### 3.4 Intestinal morphology

Table 6 shows the effects of PAR supplementation on the intestinal morphology of pre-bred laying hens. In both the duodenum and jejunum, no significant differences were observed among Groups A to F for intestinal VH, VW, CD, or the V/C ratio (*p* > 0.05). However, in the ileum, the intestinal VH of Group A was significantly lower than that of Groups E and F (*p* < 0.05). Similarly, the V/C ratio in Group A was significantly lower than that in Group E (*p* < 0.05). Furthermore, a significant linear and quadratic increase was observed in these ileal measurements with increasing proportions of PAR replacing soybean meal (*p* < 0.05). These findings collectively suggest that the incorporation of PAR as a substitute for soybean meal promotes the development of intestinal morphology to a certain extent, thereby potentially enhancing intestinal absorption.

**Table 6:** Effects of PAR on the intestinal morphology of pre-breeding laying hens.

Items		Groups						*P* value		
		A	B	C	D	E	F	SEM	Linear	Quadratic
Duodeum	VH/mm	1.59	1.72	1.63	1.41	1.51	1.53	0.05	0.234	0.497
	VW/mm	0.27	0.26	0.23	0.33	0.31	0.29	0.01	0.218	0.480
	CD/mm	0.37	0.29	0.31	0.27	0.33	0.31	001	0.366	0.154
	V/C	4.36	6.09	5.25	5.32	4.67	4.96	0.24	0.814	0.510
Ileum	VH/mm	1.21^bc^	1.15^bc^	1.17^bc^	1.09^c^	1.64^a^	1.50^a^	0.06	0.009	0.01
	VW/mm	0.20	0.23	0.21	0.23	0.18	0.24	0.01	0.657	0.903
	CD/mm	0.24	028	0.24	0.26	0.22	0.23	0.01	0.288	0.447
	V/C	5.12^bc^	4.28^c^	5.02^bc^	4.11^c^	7.48^a^	6.74^ab^	0.35	0.013	0.012
Jejunum	VH/mm	1.30	1.12	0.96	1.07	1.28	1.17	0.40	0.925	0.118
	VW/mm	024	0.28	0.29	0.24	0.25	0.23	0.01	0.458	0.459
	CD/mm	0.32	0.25	0.29	0.29	0.37	0.26	0.01	0.892	0.980
	V/C	4.20	4.54	3.42	3.77	3.51	4.58	0.19	0.831	0.257

### 3.5 Intestinal pH

Table 7 shows the impact of PAR on the gastrointestinal pH of pre-bred laying hens. In the duodenal segment, while no significant differences in luminal pH were observed among the individual treatment groups (A-F) (*p* > 0.05), a significant quadratic increase in luminal pH was noted in correlation with increasing PAR substitution levels (*p* < 0.05). Conversely, in the ileal compartment, the luminal pH of Group A was significantly greater than that of the other experimental groups (p < 0.05). Similarly, in the jejunal region, the luminal pH in Groups A through C was significantly greater than that in Group F (*p* < 0.05). For both the ileal and jejunal segments, the luminal pH significantly decreased in a linear and quadratic manner as the substitution rate of PAR increased (*p* < 0.05).

**Table 7:** Effects of PAR on the intestinal pH of pre-adult laying hens.

Items	Groups						*P* value		
	A	B	C	D	E	F	SEM	Linear	Quadratic
Duodeum	6.43^ab^	6.40^ab^	6.45^a^	6.51^a^	6.47^a^	6.34^b^	0.02	0.581	0.027
Ileum	6.85^a^	6.48^b^	6.41^b^	6.34^cd^	6.35^cd^	6.28^cd^	0.05	<0.001	<0.001
Jejunum	6.43	6.42	6.40	6.32	6.34	6.08	0.08	0.024	0.040

These findings collectively indicate that PAR exerts a regulatory effect on gastrointestinal pH, which could positively influence the digestive efficiency, nutrient absorption, and overall health of Nandan Yao chickens.

### 3.6 Analysis of the intestinal flora

**Figure 1:**
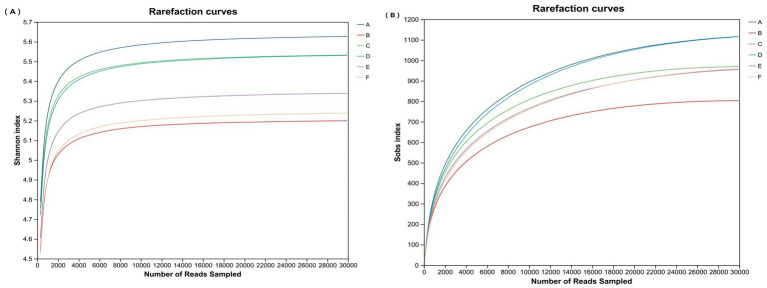
Dilution curves. **(A)** Shannon index dilution curves for each sample. **(B)** Sobs index dilution curves for each sample. Note: Peer data that share the same letter or have no letter indicate that the difference is not significant, whereas different letters indicate a significant difference (*p* < 0.05). A: 0% PAR replacing soybean meal; B: 10% PAR replacing soybean meal; C: 20% PAR replacing soybean meal; D: 30% PAR replacing soybean meal; E: 40% PAR replacing soybean meal; and F: 50% PAR replacing soybean meal.

To assess the impact of replacing soybean meal with varying percentages of PAR on the intestinal microbial community structure of pre-bred laying hens, a comprehensive analysis of the intestinal flora was conducted via high-throughput sequencing technology. This process successfully yielded a total of 1,147,854 optimized sequences from the samples. The dilution curve (Figure 1) plateaued, confirming that the volume of sequencing data was sufficient to reliably represent the composition of the intestinal flora.

To quantify the richness and diversity of the gut microbiota, various *α* diversity indices, including the Ace, Chao, Shannon, Simpson, Coverage, and Sobs indices, were utilized. As shown in Table 8, the α diversity indices of the intestinal microorganisms across Groups A to F did not significantly differ (*p* > 0.05). These findings indicate that the overall diversity of intestinal microorganisms was not substantially affected by the different PAR treatment groups.

**Table 8:** Effects of PAR on the Alphan diversity indices of the intestinal microorganisms of pre-bred laying hens.

Items	Groups						*P* value		
	A	B	C	D	E	F	SEM	Linear	Quadratic
ACE	1158.89	807.34	977.63	1163.26	992.57	1009.80	44.90	0.997	0.904
Chao	1134.60	804.03	972.12	1136.20	969.37	987.98	42.67	0.936	0.920
Shannon	5.63	5.20	5.53	5.53	5.34	5.24	0.07	0.325	0.602
Simpson	0.01	0.02	0.01	0.01	0.02	0.02	0.01	0.303	0.458
Coverage	0.997	0.999	0.997	0.997	0.998	0.998	0.0004	0.226	0.182
Sobs	1116.33	803.67	970.00	1115.33	955.33	959.67	40.61	0.834	0.939

**Figure 2:**
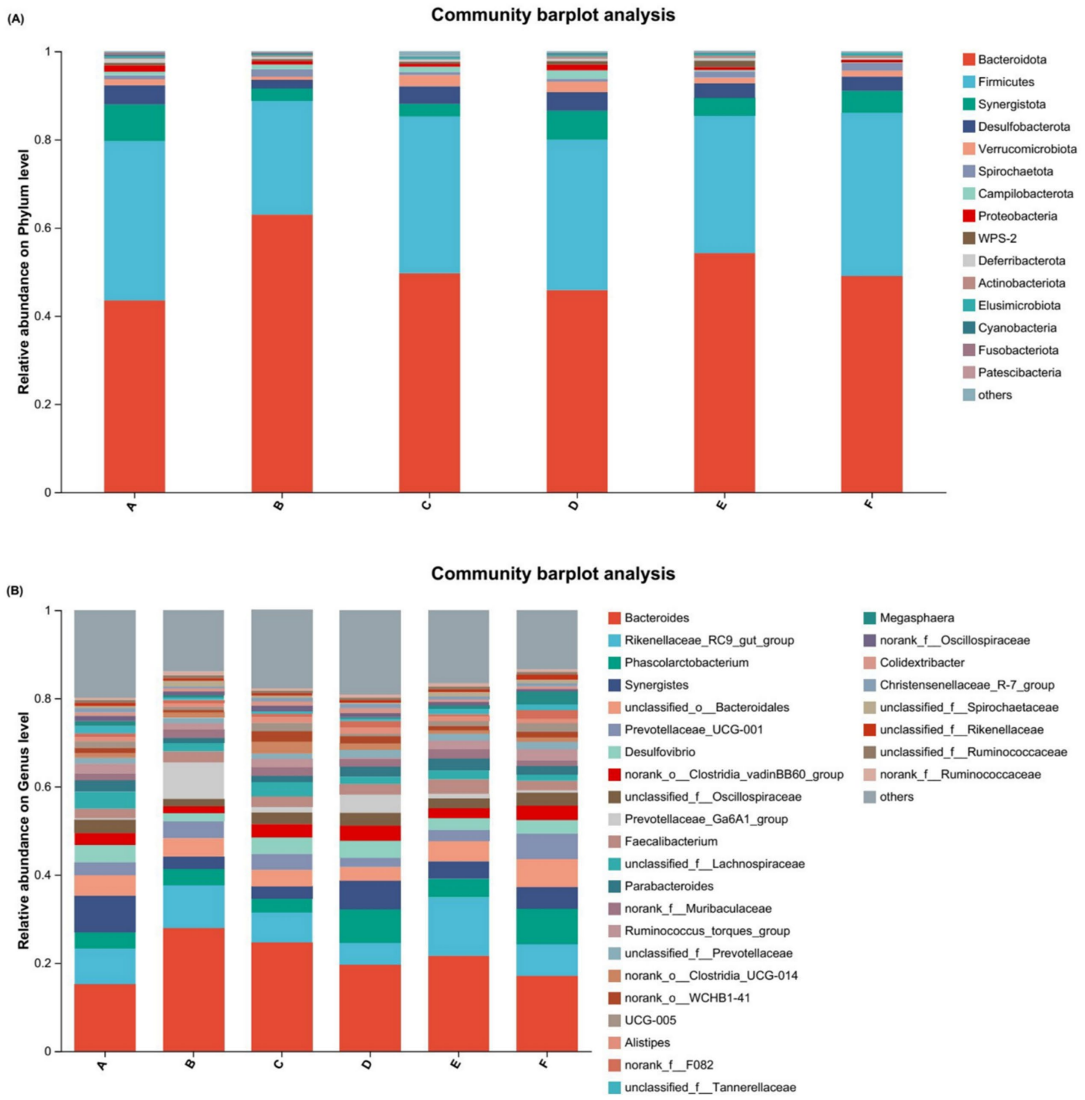
Changes in community composition of cecum microorganisms of the PAR, Blattella americana, on cecum microorganisms of pre-breeding laying hens at the phylum and genus levels of water. **(A)** Changes in species composition at the phylum level. **(B)** Changes in species composition at the genus level. Different colored lines represent different species, and the length of the line represents the size of the proportion of each species. A: 0% PAR replacing soybean meal; B: 10% PAR replacing soybean meal; C: 20% PAR replacing soybean meal; D: 30% PAR replacing soybean meal; E: 40% PAR replacing soybean meal; and F: 50% PAR replacing soybean meal.

Figure 2A shows that the predominant phyla included *Bacteroidota*, *Firmicutes*, *Synergistota*, *Desulfobacterota*, and *Verrucomicrobiota*. Furthermore, as shown in Table 9, the differences in relative abundance among the various groups at the phylum level were not statistically significant (*p* > 0.05). At the genus level, the dominant taxa, as shown in Figure 2B, included *Bacteroides*, *Rikenellaceae_RC9_gut_group*, *Phascolarctobacterium*, *Synergistes*, and *unclassified o__Bacteroidales*.

**Table 9:** Effects of PAR on the cecal intestinal flora of pre-bred laying hens.

Items	Groups						*P* value		
	A	B	C	D	E	F	SEM	Linear	Quadratic
*Bacteroidota*	43.50	62.93	49.65	45.83	54.21	49.00	2.12	0.957	0.765
*Firmicutes*	36.12	25.83	35.56	34.08	311.17	37.03	1.59	0.574	0.613
*Synergistota*	8.30	2.74	2.84	6.59	4.00	5.00	0.99	0.672	0.587
*Desulfobacterota*	4.36	2.06	4.00	4.20	3.34	3.23	0.29	0.779	0.962
*Verrucomicrobiota*	1.37	0.74	2.53	2.41	1.28	1.34	0.32	0.845	0.549
*Spirochaetota*	0.90	1.62	0.68	0.65	1.43	1.74	0.20	0.403	0.421
*Campilobacterota*	0.81	1.05	1.25	1.92	0.30	0.19	0.29	0.446	0.295
*Proteobacteria*	1.46	0.82	0.64	1.31	0.66	0.53	0.13	0.099	0.261
*WPS-2*	0.58	0.44	0.44	0.75	1.46	0.11	0.13	0.705	0.527
*Deferribacterota*	0.84	0.48	0.47	0.56	0.64	0.23	0.15	0.425	0.735
*Unclassified_k__norank_d__Bacteria*	0.29	0.27	1.09	0.39	0.30	0.35	0.13	0.908	0.529
*Actinobacteriota*	0.21	0.29	0.15	0.56	0.52	0.66	0.09	0.054	0.154
*Elusimicrobiota*	0.33	0.33	0.42	0.35	0.46	0.45	0.07	0.520	0.818
*Cyanobacteria*	0.36	0.20	0.20	0.35	0.19	0.12	0.05	0.275	0.553
*Fusobacteriota*	0.56	0.19	0.003	0.06	0.03	0.02	0.10	0.096	0.123
*Patescibacteria*	0.013	0.003	0.090	0	0.010	0	0.013	0.631	0.550

### 3.7 Transcriptome sequencing

To investigate the effects of replacing soybean meal with varying percentages of PAR on the pre-breeding intestinal function of laying hens, transcriptomic analysis, differential gene expression analysis, and functional annotation were performed.

Transcriptomic analysis outcomes: Transcriptome profiling was conducted on three samples per group, generating a robust dataset of 123.36 GB of high-quality sequence data. Each individual sample yielded more than 5.97 GB of clean data, with the proportion of Q30 bases consistently exceeding 94.82%. The analysis identified a total of 24,145 expressed genes (comprising 23,519 annotated and 626 novel genes) and 72,572 expressed transcripts (including 52,161 known and 20,411 novel transcripts).

**Figure 3:**
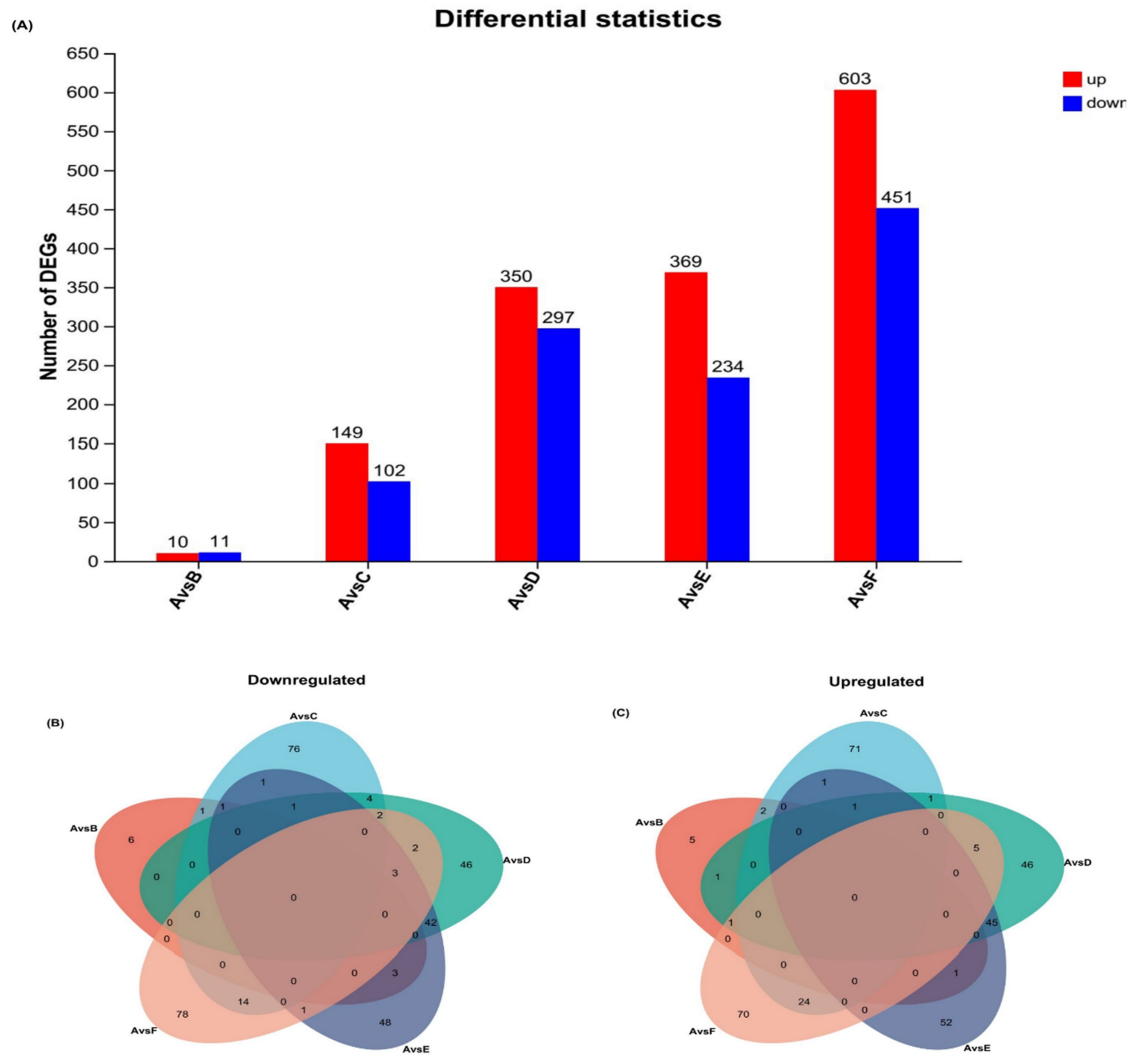
Effects of different doses of PAR on alterations in the transcriptome of jejunal DEGs in pre-bred laying hens. **(A)** Bar graphs showing the number of up- and downregulated genes in A versus B, A versus C, A versus D, A versus E, and A versus F. The number of upregulated and downregulated genes in A versus B is shown in red. Downregulated genes are shown in red, and upregulated genes are shown in blue. **(B)** Wayne plots showing the number of DEGs that were commonly or uniquely downregulated between the test and control groups A. **(C)** Wayne plots showing the number of DEGs that were commonly or uniquely upregulated between the test and control groups A. A: 0% PAR replacing soybean meal; B: PAR replacing soybean meal; C: 20% PAR replacing soybean meal; D: 30% PAR replacing soybean meal; E: 40% PAR replacing soybean meal; and F: 50% PAR replacing soybean meal.

Differential gene expression analysis: A total of 2,576 differentially expressed genes (DEGs) were identified across the six transcriptomes (Figure 3A). The number of DEGs varied significantly among the comparisons with the control group A: A vs. B: 22 DEGs (10 upregulated, 11 downregulated). A vs. C: 251 DEGs (149 upregulated, 102 downregulated). A vs. D: 647 DEGs (350 upregulated, 297 downregulated). A vs. E: 603 DEGs (369 upregulated, 234 downregulated). A vs. F: 1,054 DEGs (603 upregulated, 451 downregulated). Figures 3B,C further illustrate that each group comparison (A vs. B, A vs. C, A vs. D, A vs. E, and A vs. F) presented unique sets of downregulated DEGs, with 6, 78, 46, and 48 DEGs identified for the first four comparisons, respectively. The corresponding numbers of unique upregulated DEGs were 5, 71, 46, 52, and 70, respectively. Importantly, no shared upregulated or downregulated DEGs were observed among the treatment groups.

**Figure 4:**
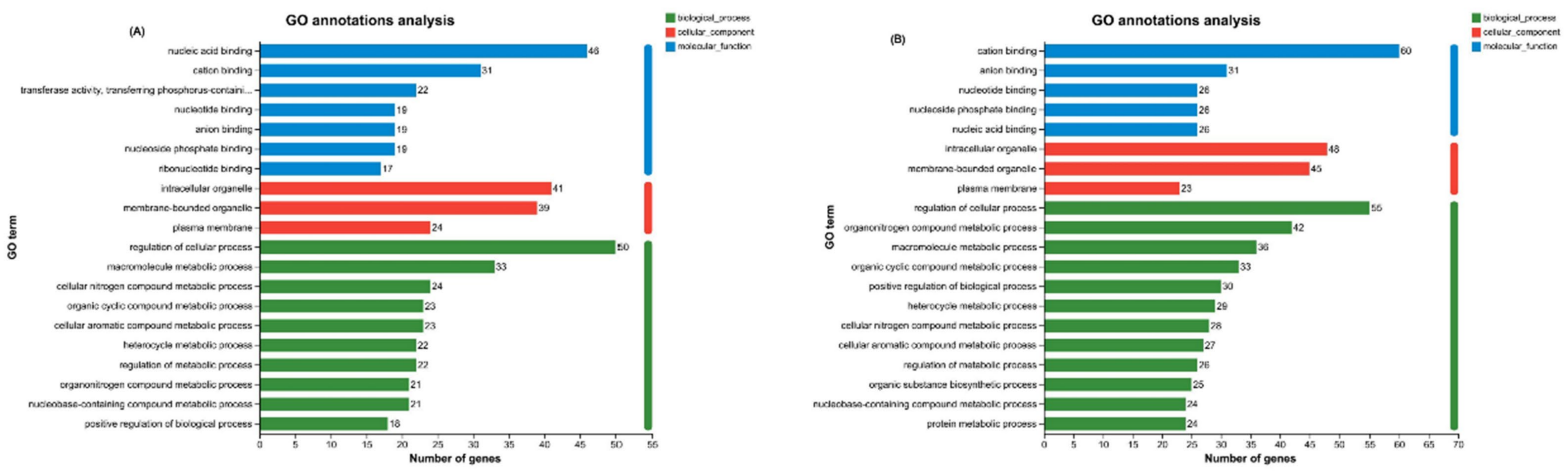
**(A)** GO functional annotation analysis of upregulated DEGs; **(B)** GO functional annotation analysis of downregulated DEGs.

Functional Annotation and Enrichment Analysis of DEGs: To elucidate the biological impact of PAR substitution, functional annotation via Gene Ontology (GO) and Kyoto Encyclopedia of Genes and Genomes (KEGG) analyses, along with enrichment analysis, were performed. The general GO and KEGG annotations (Figures 4A,B) revealed that the upregulated genes were predominantly involved in biological processes such as cell proliferation and DNA repair, were linked to ribosomal and mitochondrial functions at the CC level, and were associated with protein binding and enzymatic activity at the MF level. The downregulated genes were associated with biological processes such as apoptosis and the immune response, pertained to the cell membrane structure at the cellular component level, and were linked to signaling functions at the molecular function level.

**Figure 5:**
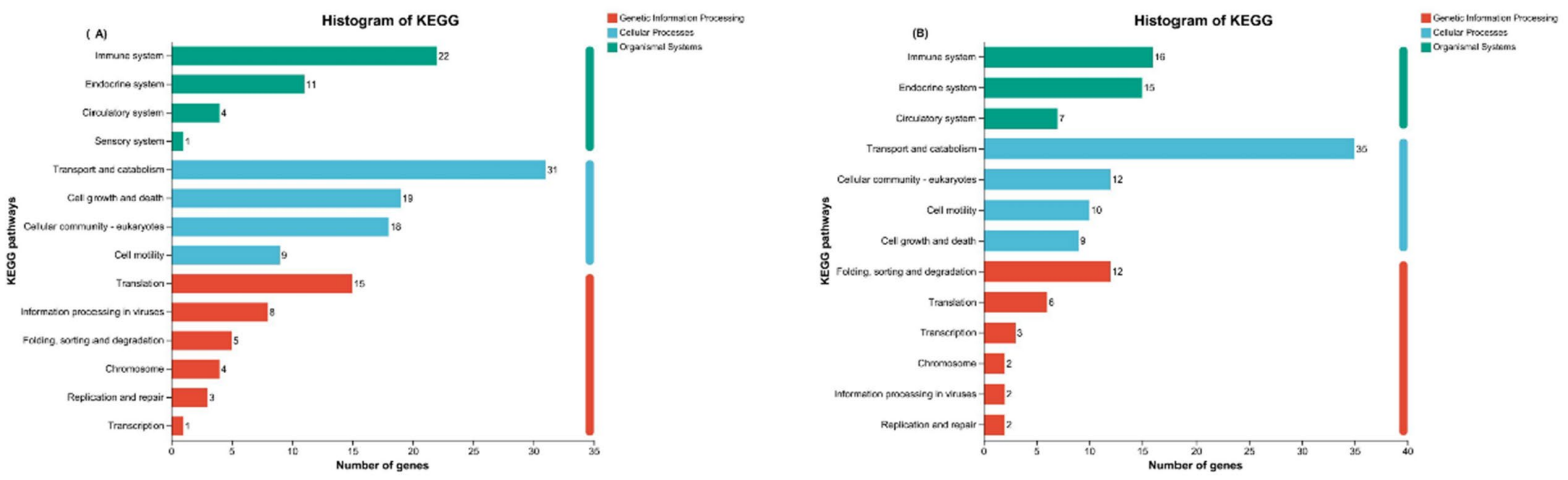
**(A)** KEGG functional annotation analysis of upregulated DEGs; **(B)** KEGG functional annotation analysis of downregulated DEGs.

KEGG pathway analysis (Figures 5A,B) revealed that the upregulated genes were involved mainly in pathways related to the immune response, endocrine regulation, material transport and metabolism, and cell proliferation and apoptosis. The downregulated genes were involved in pathways related to material transport and metabolism, the immune response, the endocrine system, and cellular motility.

**Figure 6:**
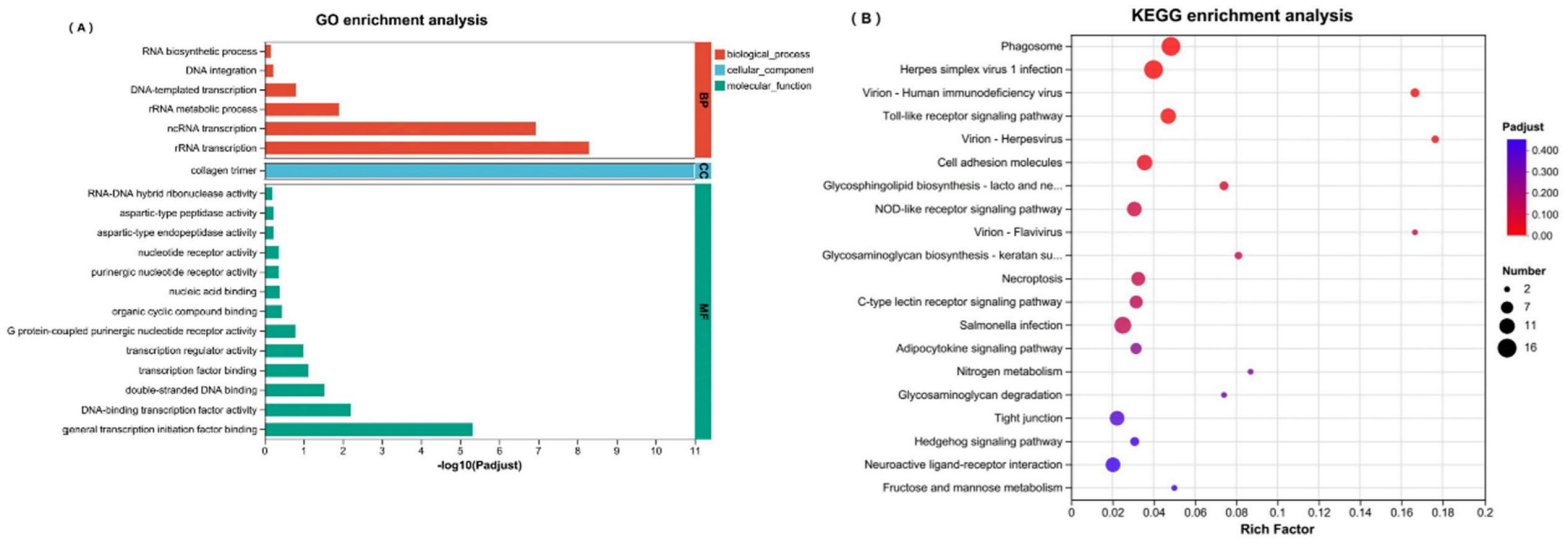
**(A)** GO enrichment analysis of upregulated DEGs; **(B)** KEGG enrichment analysis of upregulated DEGs.

GO and KEGG enrichment analyses revealed enrichment of upregulated genes (Figures 6A,B) and the results highlighted their pivotal roles in biological processes, particularly RNA biosynthesis, DNA repair, and transcriptional activation. At the molecular functional level, gene products exhibit ribonuclease activity and nucleic acid binding capacity. KEGG pathway analysis revealed significant enrichment in various biological pathways, including viral infection pathways (e.g., human herpesvirus and flavivirus infection), cellular immune response pathways (e.g., Toll-like receptor and NOD-like receptor signaling pathways), and pathways related to cell adhesion molecule biology and metabolism.

**Figure 7:**
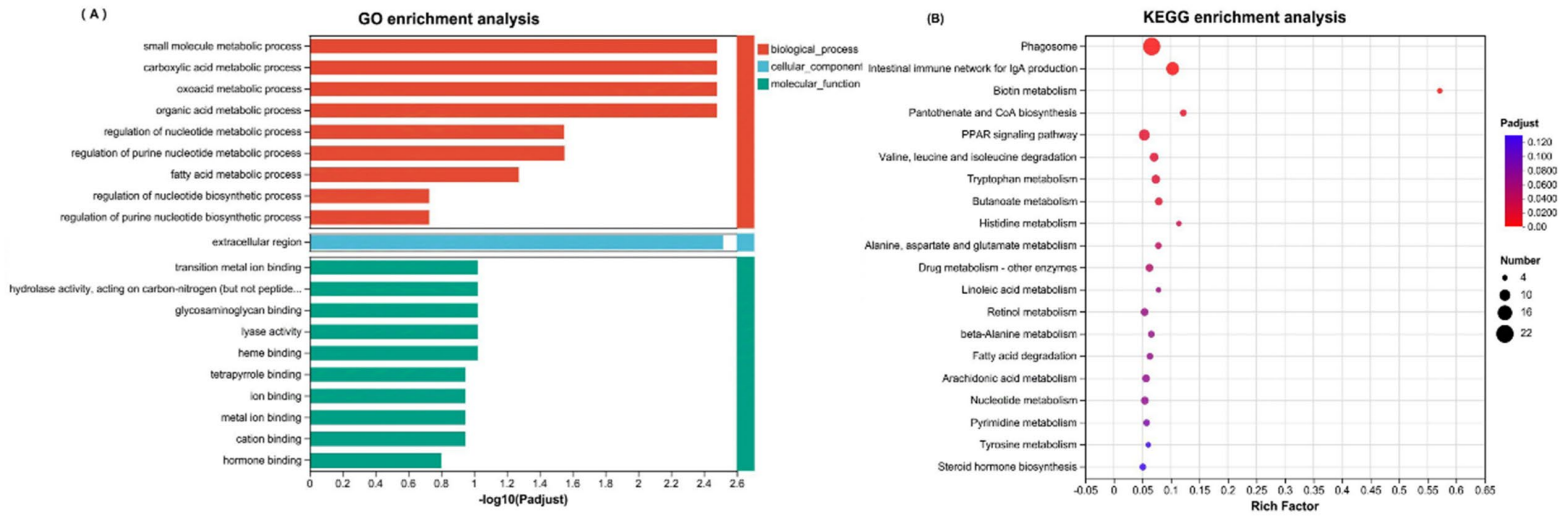
**(A)** GO enrichment analysis of downregulated DEGs; **(B)** KEGG enrichment analysis of downregulated DEGs.

Enrichment of downregulated genes (Figures 7A,B) GO enrichment revealed their involvement primarily in the metabolism of small molecules and organic acids in biological processes, with cellular component associations related to extracellular region activities and molecular functions linked to the binding of transition metal ions. KEGG analysis revealed crucial roles in diverse metabolic pathways, such as the phagosomal pathway, biotin metabolism, coenzyme A biosynthesis, and the PPAR signaling pathway.

## 4 Discussion

### 4.1 The viability of PAR for avian husbandry

PAR, sourced from a supply chain adhering to stringent Good Agricultural Practice standards, exhibits an exceptional baseline safety profile because of its rigorous front-end control over critical risk factors such as microbial loads and heavy metals (14). With a high crude protein content of 73.41%, PAR shows significant potential as a substitute for conventional protein sources such as soybean meal and fishmeal, pioneering a “pharma-feed” circular economy model that bridges the biomedical and modern agricultural sectors.

Its application value is particularly high in poultry production, as its nutritional composition meets poultry’s inherent demand for high-protein feed. Additionally, its chitin content offers prebiotic functions, aligning with the global trend toward antibiotic-free farming (5). This viability is supported by poultry’s natural adaptation to insect protein, as their digestive systems are well suited for it, a fact corroborated by studies confirming the high digestive capacity of poultry fluids for insect protein (15). Consequently, PAR is presented not only as a cost-effective protein source but also as a functional feed ingredient capable of enhancing intestinal health and mitigating farming risks, offering a strategic competitive advantage.

However, despite its inherent safety, comprehensive evaluation through systematic poultry feeding trials and toxicological studies is crucial to address potential risks such as residual allergens (e.g., tropomyosin), microbial contamination (e.g., *Salmonella*) during storage and transport, solvent residues, and endogenous toxins (16). These investigations are essential for scientific rigor and for securing market access from regulatory bodies in major economies such as the EU, the US, and China. Demonstrating efficacy through comprehensive data will be pivotal for alleviating market concerns and ensuring farmer acceptance.

In conclusion, PAR presents a compelling and feasible option for poultry farming, offering superior safety, high nutritional value comparable to that of fishmeal, and prebiotic functions that support antibiotic-free production. Its potential is further bolstered by poultry’s natural feeding behavior and scientific validation. Nevertheless, its full realization is contingent upon systematic risk assessment and stringent regulatory approval. Successfully navigating these hurdles would position PAR as an innovative protein source and a key driver for the green and efficient development of the poultry industry, providing substantial economic and social value.

### 4.2 PAR improved the growth performance of laying hens in the pre-breeding period

Insect protein powder has been strategically incorporated into poultry feed formulations as an innovative and sustainable alternative to conventional animal protein sources (17). Recent scientific evidence indicates that integrating insect protein powder can not only maintain but also surpass the performance of traditional protein feeds, providing essential amino acids, exceptional nutrient digestibility, and high feed acceptability (10, 18). Moreover, insect protein powder is rich in bioactive compounds such as antimicrobial peptides and chitosan, which significantly enhance digestion, optimize intestinal physiological functions, rebalance the gut microbiota, and bolster immune defenses (19).

In poultry production, critical metrics such as daily weight gain, daily feed intake, and the feed-to-weight ratio are used to evaluate growth efficiency and economic viability (20). Previous studies have demonstrated the positive effects of PAR: Long et al. reported that supplementing feed with 1% and 2% PAR powder improved growth performance and moderately enhanced immune responses in mice (11). The same authors reported that substituting 50% to 75% of traditional fishmeal with PAR did not adversely affect feed utilization, antioxidant capacity, or immune function in juvenile Nile tilapia (10). Similarly, Al-Salhie et al. (18) revealed that PA protein could fully replace conventional protein sources in quail without compromising production performance and, under specific conditions, even significantly elevated growth performance, highlighting its potential in high-quality poultry nutrition.

In this study, PAR was employed as a partial substitute for soybean meal in the basal diet of pre-bred laying hens. While no statistically significant differences in overall growth performance were observed between the experimental and control groups, the average daily weight gain and feed-to-weight ratio were notably greater in sample groups B, C, E, and F than in control group A. These findings align with those of Long et al. and suggest that replacing soybean meal with PAR in the pre-breeding diet of laying hens did not impair growth performance. Specifically, the experimental groups with 10%, 40%, and 50% PAR substitution ratios presented superior performance, underscoring the efficacy of PAR as a sustainable protein source in poultry nutrition.

### 4.3 PAR increased the levels of intestinal immune factors in laying hens in the pre-breeding period

The intestinal immune barrier, which comprises gut-associated lymphoid tissue, resident immune cells, and their secretory products, such as immunoglobulins (IgA, IgG, and IgM) and various cytokines, serves as a critical first line of defense (21). This system is responsible for producing approximately 70% to 80% of the body’s immunoglobulins, which are indispensable for preventing pathogen invasion, maintaining gut microbiota equilibrium, reinforcing intestinal mucosal integrity, modulating immune responses, and facilitating nutrient absorption (22, 23).

Previous investigations have provided empirical support for the immunomodulatory potential of dietary interventions. For example, Zhang et al. reported an elevation in serum immunoglobulin levels in dairy goats following specific dietary inclusion (24), and Jiang demonstrated that supplementing broiler chicken feed with PA powder markedly increased their serum immunoglobulin concentrations (25). More recently, Ou et al. (9) established that the strategic incorporation of PAR into the diet can effectively enhance both immune function and antioxidant capacity in Sanhuang chickens. The findings of this study corroborate these earlier observations. In our investigation, a discernible trend emerged: as the proportion of PAR substituted for soybean meal increased, the levels of IgA and IgG within the duodenum and jejunum, along with the IgM in these same intestinal segments, generally tended to increase. Furthermore, the relationship between the level of PAR substitution and the observed increases in IgA and IgM displayed a significant quadratic characteristic, suggesting a potentially nonlinear dose–response effect.

These results strongly suggest that the use of PAR as a replacement for soybean meal in the diet of Nandan chickens not only preserves the integrity of their intestinal immunoglobulin levels but also appears to confer a degree of immunostimulatory benefit, thereby enhancing their overall immune function. This highlights the potential of insect-based proteins such as PAR to positively influence gut immunity in poultry.

### 4.4 PAR increased the levels of intestinal cytokine factors in laying hens in the pre-breeding period

Within the intricate regulatory network governing the immune system, cytokines such as TNF-*α*, IFN-*γ*, IL-2 function as pivotal signaling molecules, orchestrating critical immune responses and modulating inflammatory processes (26). TNF-α plays a key role in acute inflammation and autoimmune diseases (27), IFN-γ amplifies cell-mediated immunity by stimulating macrophages and natural killer cells (28), and IL-2 is central to immune regulation and homeostasis and is synthesized primarily by activated T cells (29, 30).

The findings of this study provide compelling evidence that substituting soybean meal with PAR in the feed formulation significantly elevated the intestinal levels of these key cytokines—TNF-α, IL-2, and IFN-γ—in growing laying hens. This observed increase suggests that PAR supplementation effectively stimulates the local intestinal immune environment. Consequently, the utilization of PAR as a replacement for soybean meal not only maintains but also potentially enhances the intestinal cytokine milieu in pre-breeding laying hens. This immunomodulatory effect is posited to contribute meaningfully to the overall enhancement of poultry immune competence, positioning PAR as a promising feed ingredient for bolstering gut health and immunity.

### 4.5 PAR improved the intestinal morphology of laying hens in the pre-breeding period

The structural integrity of the gut is paramount for optimal health, serving as the primary site for nutrient digestion and absorption and as the central hub of the immune defense system in chickens (31, 32). Morphological parameters of the intestinal epithelium—VH, CD, VW, and the V/C—are the established indicators of nutrient assimilation efficiency and gut health (33, 34). A reduced VW often correlates with enhanced secretory activity, whereas an elevated V/C ratio indicates increased absorptive surface area and improved digestive capacity; conversely, a diminished ratio suggests compromised absorption (35, 36). Therefore, precise measurement of these parameters in laying hens provides a valuable metric for assessing intestinal growth and development, offering a robust theoretical foundation for optimizing feeding strategies (37).

Previous investigations have demonstrated the positive impact of insect-based proteins on gut morphology. Ou et al. reported that substituting 40% expanded soybean meal enhanced the intestinal morphology of Sanhuang chickens (9). Furthermore, Zhou et al. reported that insect protein powder could repair compromised intestinal mucosa and chorioallantoic villi in a murine model of pathological damage (38). Based on these findings, Zhang et al. confirmed that incorporating insect protein ingredients bolstered intestinal immune cell activity and reduced intestinal inflammation (24). Given the intrinsic link between rapid chicken growth and digestive tract integrity, evaluating these parameters is critical (38).

In this study, we analyzed the duodenum and jejunum of laying hens and found no statistically significant differences in VH, VW, CD, or the V/C ratio across different dietary groups in these regions. However, a distinct pattern emerged in the ileum. Compared to Groups E and F, Group A (the control diet) presented significantly lower ileal VH, which likely incorporated higher levels of PAR. This observation is significant, as the ileal VH demonstrated a linear and quadratic increase that was correlated with a greater proportion of soybean meal being replaced by cockroach meal. Additionally, the V/C ratio in Group A was significantly lower than that in Group E, which aligns with the results reported by Ou et al. (9). Collectively, these results strongly suggest that substituting soybean meal with PAR in the basal rations does not negatively affect the intestinal morphology of prereading laying hens. More specifically, a substitution ratio of 40% appears to confer a significant beneficial effect, notably enhancing ileal structural integrity, which is likely to positively influence overall gut function and health.

### 4.6 PAR modulates the intestinal pH of laying hens during the pre-breeding period

The gut pH is a crucial physiological indicator of gut health and productivity, significantly influencing nutrient absorption, digestive enzyme activity, and the balance of microbial communities in laying hens (39). An optimal acidic environment fosters beneficial bacteria (e.g., *Lactobacillus* and *Bifidobacterium*) while inhibiting pathogens (e.g., *Escherichia coli* and *Salmonella*), thereby promoting digestion, absorption, and overall animal health (40). This regulation of the gut pH is vital for ensuring healthy growth, improving feed conversion, and increasing disease resistance in layer breeding (41, 42).

This study investigated the impact of the substitution of PAR (on the gut pH of laying hens). The results revealed a linear and quadratic relationship between PAR inclusion and duodenal pH. Notably, the ileal pH significantly decreased in all the groups with PAR addition, and both the ileal and jejunal pH exhibited linear and quadratic reductions with increasing PAR substitution ratios. These lower intestinal pH values offer several advantages: enhanced digestive enzyme activity; an acidic environment facilitates the operation of digestive enzymes (e.g., pepsin and trypsin) at peak efficiency, augmenting the digestion and absorption of proteins and carbohydrates; and the inhibition of pathogenic microbial growth. The acidic milieu suppresses the proliferation of pathogenic microorganisms, mitigating the risk of intestinal infections and preserving gut health.

Promotion of nutrient absorption: An optimal acidic environment aids in the dissolution and absorption of nutrients, including minerals and vitamins, thereby increasing nutrient bioavailability.

These findings suggest that PAR has the potential to modulate the intestinal pH of poultry, optimize the gut environment, and increase the feed conversion efficiency of livestock and poultry. This discovery offers a novel perspective for managing gut health and refining feed formulations.

### 4.7 PAR maintains the balance of the intestinal flora in laying hens during the pre-breeding period

The gut represents a finely tuned and complex microbial ecological habitat, where numerous microbial populations have coevolved synergistically with host cells and the immune system (43, 44). Its equilibrium is fundamental to host health, regulating crucial life activities such as nutrient digestion, metabolic processes, and immune functions (45). Alpha diversity indices, including the Ace, Chao (estimating total species richness), Shannon (reflecting diversity and evenness), Simpson (focusing on species dominance), Coverage (measuring detected species proportion), and Sobs (observed species richness) indices, are vital for assessing microbial biodiversity.

In this study, replacing soybean meal with PAR had no significant effect on the alpha diversity index in the jejunum of reared laying hens. These findings suggest that PAR maintains the biodiversity, complexity, and stability of the gut microbial community, ensuring nutrient intake and health during a critical growth period.

Analysis of species composition at the phylum level revealed the predominant presence of *Bacteroidota*, *Firmicutes, Synergistota*, *Desulfobacterota*, and *Verrucomicrobiota. Bacteroidetes* and *Firmicutes* are core poultry flora (46) that play key roles in nutrient digestion [the *Bacteroidota* facilitates fermentation (47)], the innate immune response [*Firmicutes* synthesizes fatty acids (48, 49)], the regulation of intestinal diseases, the inhibition of harmful bacteria, and the production of short-chain fatty acids. *Synergistota* may support catabolism of poorly utilized substrates and regulate the gut microenvironment, whereas *Verrucomicrobiota* is associated with gut health and anti-inflammatory properties (50). However, *Desulfobacterota* has been linked to harmful effects, including obesity (51).

At the genus level, the predominant flora included *Bacteroides*, *Rikenellaceae_RC9_gut_group*, *Phascolarctobacterium*, and *Synergistes*, which differed from some other reported chicken gut flora compositions (52). *Phascolarctobacterium* is a beneficial gut bacterium that produces propionic acid, contributing to the energy supply, gut health, and pathogen suppression. *Rikenellaceae_RC9_gut_group* is involved in structural carbohydrate degradation (53) and has been positively correlated with meat fat content in Tan sheep (54, 55).

These findings indicate that the introduction of PAR as a dietary ingredient did not adversely affect the abundance or diversity of intestinal microorganisms in pre-bred laying hens. Instead, it effectively maintains the stability and advanced complexity of the intestinal flora, fostering a favorable microbial environment to support growth and development. These results further confirm the potential application value of PAR in the laying chicken farming industry, providing a scientific basis for promoting green and sustainable farming techniques.

### 4.8 Effects of the use of PAR as a substitute for soybean meal on the intestinal transcriptome of laying hens during the pre-breeding period

Transcriptome sequencing (RNA-Seq) was employed to analyze jejunal gene expression in pre-breeding laying hens following the ingestion of different doses of PAR, identifying a total of 2,576 differentially expressed genes (DEGs). This approach aimed to elucidate the molecular mechanisms by which PAR influences intestinal health and function, given the crucial role of the intestine in nutrient digestion, absorption, and immune defense (56–58).

KEGG enrichment analysis of the upregulated DEGs revealed significant enrichment in pathways related to viral infection (e.g., human herpesvirus and flavivirus infection); cellular immune responses (involving the Toll-like receptor and NOD-like receptor signaling pathways); and the biosynthesis and metabolism of cell adhesion molecules.

These findings suggest that PAR significantly impacts pathogen defense, immune regulation, and cellular communication, particularly by enhancing antiviral and immune signaling. The key upregulated genes involved in gut health-related pathways included those regulating inflammatory and antiviral responses (IRF9, NFKBIE, and STAT1), intestinal barrier function (CLDN23, MID1IP1, and MOBP), immune defense against pathogens (LYZ, CYBB, and CD14), intestinal metabolic and developmental processes (GFOD1, CECR1, and CECR2), immune regulation and inflammation control (TDO2, IFI27L2, and CCL19), and immune cell recruitment/activation (TNFRSF13C, IL2RA, P2RY2, IL22RA2, CD1G, and CCR8) (59–73).

Conversely, KEGG enrichment analysis indicated that the downregulated DEGs play important roles in several metabolic pathways, including the phagosomal pathway.

Biotin metabolism; Coenzyme A biosynthesis; PPAR signaling pathway. The major downregulated genes included FABP4 (adipocyte protein 2, involved in metabolic diseases and interacting with PPARC1A), ALDH5A1 (succinate semialdehyde dehydrogenase, linked to oxidative stress), TRPA1 and KCNS3 (regulating gut sensory and motor functions), MMP13 and HEPACAM2 (involved in extracellular matrix remodeling and tissue repair), LMBRD2 and BST1 (associated with mitochondrial metabolism, energy production, and lysosomal function), and CIP2A (regulating the cell cycle and intestinal regeneration) (74–82).

Collectively, these findings reveal the multifaceted effects of PAR on the intestinal health of laying hens, demonstrating its ability to modulate immunomodulation, enhance barrier function, and influence metabolic homeostasis at the molecular level.

## 5 Limitations and future perspectives

This study’s experimental design was confined to an 11-week rearing period, thereby precluding a comprehensive assessment of the long-term impact of PAR on laying performance (e.g., egg production rate and egg quality) or meat quality. Furthermore, critical practical considerations such as consumer acceptance, palatability, and the environmental footprint of large-scale insect farming have not been adequately addressed. While safety evaluations encompassed heavy metals and microorganisms, they did not delve into allergens or drug residues or comply with international standards such as those set by the EU EFSA/FDA.

Consequently, future investigations are imperative to extend the experimental duration to the laying stage, systematically evaluating the effects of PAR on reproductive performance, egg quality, and the organoleptic properties of meat. Concurrently, cross-cultural consumer surveys and palatability optimization strategies should be pursued, alongside the quantification of the PAR supply chain’s life cycle environmental benefits and economic viability. Further rigorous safety assessments, including allergen thermal stability and tissue residue monitoring, are essential to satisfy regulatory mandates. Ultimately, exploring its trans-species applicability in other poultry or aquaculture systems will facilitate the holistic transition of PAR from laboratory inquiry to sustainable industrial practice.

## 6 Conclusion

This study revealed that an appropriate substitution of PAR for soybean meal improved the immune function pH and intestinal morphology of laying hens in the pre-breeding period without affecting their growth performance or cecum flora composition. Transcriptome analysis further revealed the multidimensional effects of PAR on intestinal health, including immunomodulation, barrier function and metabolic homeostasis. These findings suggest that the use of an appropriate amount of PAR to replace soybean meal is feasible. This not only alleviates the tension of protein resources but also provides a scientific basis for the resourceful treatment of insects and other wastes. Nevertheless, the potential effects of this substitution method on the nutrient composition and flavor of meat have not been explored, which is still an area that needs to be studied in depth.

## Data Availability

The datasets presented in this study are available in the NCBI BioProject repository under accession number PRJNA1232861 (https://www.ncbi.nlm.nih.gov/bioproject/?term=PRJNA1232861).

## Footnotes

^1^ https://cloud.majorbio.com

## References

[1] Smetana S Schmitt E Mathys A. Sustainable use of *Hermetia illucens* insect biomass for feed and food: attributional and consequential life cycle assessment. Resour Conserv Recycl. (2019) 144:285–96. doi: 10.1016/j.resconrec.2019.01.042

[2] Huis AV Itterbeeck JV Klunder H Mertens E Halloran A Muir G . Edible insects: Future prospects for food and feed security Rome Food and Agriculture Organization of the United Nations (2013).

[3] Acosta-Estrada BA Reyes A Rosell CM Rodrigo D Ibarra-Herrera CC. Benefits and challenges in the incorporation of insects in food products. Front Nutr. (2021) 8:687712. doi: 10.3389/fnut.2021.687712, PMID: 34277684 PMC8277915

[4] Lei SF. 1.5 percentage points: where does the potential come from?—interpretation of the "three-year action plan for reducing and replacing soybean meal in feed". Swine Industry Observation. (2023) 3:13–4. Available online at: https://kns.cnki.net/kcms2/article/abstract?v=DJp2nd4LPS09952ZdCupkLbEpY4iDTmYB4WHoTcFMVlKU4owSwBKF6f5PicZzD3Wh41UuWgwcKb7KR_6kJtAm_ajVS91tYym738KnOLvQnWSb8maNCmjB2S5r5PyAyElwjgvbmNX7mH3_gipglu8tY5mjpfW6zp9B5pw22dKUrvbCj9h1_vj4E8YkjRKhdt&uniplatform=NZKPT&language=CHS

[5] Zhang H Yao Q Wu M. Chemical composition and pharmacological activity of American cockroach. Adv Anim Med. (2018) 39:107–10. doi: 10.16437/j.cnki.1007-5038.2018.03.021

[6] Lu XX Zhu L Wu H Zhang CG Yang YY Cheng ZP . Chemical composition analysis and multicomponent determination of cockroach extracts from different farms. J Dali Univ. (2023) 8:30–8. doi: 10.3969/j.issn.2096-2266.2023.08.007

[7] Yang X Gao Z Fu J. Efficacy of Kangfuxin liquid retention enema in preventing and treating radiation-induced proctitis in cervical cancer patients: a randomized, open-label, phase III study. Int J Radiat Oncol Biol Phys. (2024) 120:S25. doi: 10.1016/j.ijrobp.2024.07.030

[8] Wu JX Niu RX Huang XQ Tian KL Dong CL. Experimental study on the anti-aging effect of cardiovascular dragon injection. J Guiyang Med Coll. (2002) 2:125–7. doi: 10.19367/j.cnki.1000-2707.2002.02.010

[9] Ou G Zhao Y Wang P Tao S Li H Zhao T. The American cockroach (*Periplaneta americana*) residue could partially replace the dietary puffed soybean meal in the three-yellow chickens. Poult Sci. (2024) 103:103967. doi: 10.1016/j.psj.2024.103967, PMID: 38941789 PMC11261138

[10] Long J S S T Z. Effects of drug residues from American cockroaches on the growth performance and immune function of mice. Heilongjiang Anim Husb Vet Med. (2022) 23:91–3. doi: 10.13881/j.cnki.hljxmsy.2022.01.0005

[11] Long X Yang W Gu Y Xiao P Yang Y Deng J. The American cockroach (*Periplaneta americana*) residue could partially replace the dietary fish meal in the juvenile Nile tilapia (*Oreochromis niloticus*). Aquac Rep. (2024) 35:101942. doi: 10.1016/j.aqrep.2024.101942

[12] Liao QH Yang ZL Sun TT Zou LQ Jiang SQ Yang XR . Effect of distance length of Nandan scallop roosters on slaughtering performance. Guangxi Anim Husb Vet Med. (2023) 39:147–9. doi: 10.3969/j.issn.1002-5235.2023.04.001

[13] Shi XT H Q Gao YR Ma QG Zhao LH Zhang JY . Effects of different levels of tryptophan on growth performance and serum biochemical indices of lightweight laying hens in the prebreeding period. Chin J Anim Husb. (2022) 58:158–62. doi: 10.19556/j.0258-7033.20210611-05

[14] Yu X Zong X Li H Wang L Zhao X Wang D . Application research on the evaluation system of group standards for traditional Chinese medicine: a case study of 90 group standards for Chinese materia medica. Zhongyi jichu yanjiu. (2024) 30:71–5. doi: 10.3969/j.issn.1006-3250.2024.01.014

[15] Kovitvadhi A Chundang P Pliantiangtam N Thongprajukaew K Tirawattanawanich C Suwanasopee T . Screening of in vitro nutrient digestibility coefficients of selected insect meals in broiler chickens, black-meat chickens and quails. J Anim Physiol Anim Nutr. (2021) 105:305–15. doi: 10.1111/jpn.13451, PMID: 32935384

[16] Gaugitsch H. Risk assessment and the challenges of biosafety management: The Austrian experience. International forum on biosafety symposium. Beijing: Ministry of Environmental Protection of the People's Republic of China, (2008).

[17] Elahi U Wang J Ma Y Wu SG Wu J Qi GH . Evaluation of yellow mealworm meal as a protein feedstuff in the diet of broiler chicks. Animals. (2020) 10:224. doi: 10.3390/ani10020224, PMID: 32019216 PMC7070689

[18] Al-Salhie KCK Al-Hummod SKM Jaber FN. Effects of the use of different levels of American cockroach (*P. americana*) powder on the productive and physiological performance of Japanese quail (*Coturnix japonica*). IOP Conf Ser Earth Environ Sci. (2021) 777:012003. doi: 10.1088/1755-1315/777/1/012003

[19] Ayman K Abo-Elezz S Refaey M. Could insect products provide a safe and sustainable feed alternative for the poultry industry? A comprehensive review. Animals. (2023) 13:1534. doi: 10.3390/ani13091534 37174571 PMC10177474

[20] Ajaykumar R Harishankar HK Rangasami SR Saravanakumar V Yazhini G Rajanbabu V . Growth performance, quantitative analysis and economics of broiler chickens as influenced by herbal dietary additives as alternative growth booster. Indian J Anim Res. (2024) 58:1139–47. doi: 10.18805/ijar.B-1462

[21] Li L Lv X He J Zhang L Li B Zhang X . Chronic exposure to polystyrene nanoplastics induces intestinal mechanical and immune barrier dysfunction in mice. Ecotoxicol Environ Saf. (2024) 269:115749. doi: 10.1016/j.ecoenv.2023.115749, PMID: 38039854

[22] Rajput M Momin T Singh A Banerjee S Villasenor A Sheldon J . Determining the association between gut microbiota and its metabolites with higher intestinal immunoglobulin A response. Vet Anim Sci. (2023) 19:100279. doi: 10.1016/j.vas.2022.100279, PMID: 36533218 PMC9755367

[23] Jin Y Lv H Wang M Cho CS Shin J Cui L . Effect of microencapsulation of egg yolk immunoglobulin Y by sodium alginate/chitosan/sodium alginate on the growth performance, serum parameters, and intestinal health of broiler chickens. Anim Biosci. (2023) 36:1241–53. doi: 10.5713/ab.23.0015 36915923 PMC10330971

[24] Zhang J Zhang M Cai W Li H Yang H Yan J . Effects of adding American cockroach powder to feed on growth performance of dairy goats. Gansu Animal Husband Vet Med. (2019) 49:25–7. doi: 10.15979/j.cnki.cn62-1064/s.2019.12.009

[25] Zhou ZR. Effects of American cockroach, Blattella americana, on the production performance of male broilers and its mechanism of action. Fuzhou: Fujian Agriculture and Forestry University (2008).

[26] Jang DI Lee AH Shin HY Song HR Park JH Kang TB . The role of tumor necrosis factor alpha (TNF-α) in autoimmune disease and current TNF-α inhibitors in therapeutics. Int J Mol Sci. (2021) 22:2719. doi: 10.3390/ijms22052719, PMID: 33800290 PMC7962638

[27] Horiuchi T Mitoma H Harashima SI. Transmembrane TNF-α: structure, function and interaction with anti-TNF agents. Rheumatology. (2010) 49:1215–25. doi: 10.1093/rheumatology/keq107 20194223 PMC2886310

[28] Abood WN I F Abdulla MA Ismail S. Immunomodulatory effect of an isolated fraction from *Tinospora crispa* on intracellular expression of INF-γ, IL-6 and IL-8. BMC Complement Altern Med. (2014) 14:1–12. doi: 10.1186/1472-6882-14-447 24969238 PMC4227069

[29] Damoiseaux J. The IL-2–IL-2 receptor pathway in health and disease: the role of the soluble IL-2 receptor. Clin Immunol. (2020) 218:108515. doi: 10.1016/j.clim.2020.108515, PMID: 32619646

[30] Dragica J Song M Wang L Zhang Y. Roles of IFN-γ in tumor progression and regression: a review. Biomark Res. (2020) 8:49. doi: 10.1186/s40364-020-00228-x 33005420 PMC7526126

[31] Pourabedin M Xu Z Baurhoo B Chevaux E Zhao X. Effects of mannan oligosaccharide and virginiamycin on the cecal microbial community and intestinal morphology of chickens raised under suboptimal conditions. Can J Microbiol. (2014) 60:255–66. doi: 10.1139/cjm-2013-0899, PMID: 24766220

[32] Sittiya J Yamauchi K Nimanong W Thongwittaya N. Influence of levels of dietary fiber sources on the performance, carcass traits, gastrointestinal tract development, fecal ammonia nitrogen, and intestinal morphology of broilers. Braz J Poult Sci. (2020) 22:eRBCA-2019-1151. doi: 10.1590/1806-9061-2019-1151

[33] Chu JH Weng TS Huang TW. The effects of replacing fish meal protein with black soldier fly meal and sodium butyrate supplementation on the growth performance, lipid peroxidation, and intestinal villi status of jade perch, *Scortum barcoo* fingerlings. Fishes. (2023) 8:437. doi: 10.3390/fishes8090437

[34] Farahat M Ibrahim D ATY K Abdallah HM Hernandez-Santana A Attia G. Effect of cereal type and plant extract addition on the growth performance, intestinal morphology, cecal microflora, and gut barriers gene expression of broiler chickens. Animal. (2021) 15:100056. doi: 10.1016/j.animal.2020.100056 33573933

[35] Zhang C Hao E Chen X Huang C Liu G Chen H . The dietary fiber level improved the growth performance, nutrient digestibility, and immune and intestinal morphology of broilers from days 22 to 42. Animals. (2023) 13:1227. doi: 10.3390/ani13071227 37048483 PMC10093110

[36] Wang L Moore DC Huang J Wang Y Zhao H Jackson CL . SHP2 regulates the development of the intestinal epithelium by modifying OSTERIX+ crypt stem cell self-renewal and proliferation. FASEB J. (2021) 35:e21106. doi: 10.1096/fj.202001091R 33165997 PMC12182962

[37] Kou Y Sun C Sun Y Liu T Wang D. Injurious effects of perfluorooctanoic acid on liver and duodenum of broiler chickens. Chin Vet J. (2024) 60:9–17. doi: 10.27109/d.cnki.ghbnu.2022.000530

[38] Zhou ZR Liu Z. Effects of cockroach, *Blattella americana*, on immune function and antioxidant capacity of broiler chickens. J Fujian Agric For Univ. (2009) 38:175–80. doi: 10.13323/jcnkijfafu(natsci)2009.02.018

[39] Pang Y Applegate TJ. Effects of dietary copper supplementation and copper source on digesta pH, calcium, zinc, and copper complex size in the gastrointestinal tract of the broiler chicken. Poult Sci. (2007) 86:531–7. doi: 10.1093/ps/86.3.531, PMID: 17297166

[40] Liu W L H W Z Wei Q Feng RZ Zhao X . Effects of different contents of oil camphor leaves in diets on intestinal pH, cecum fermentation and cecum flora of meat rabbits. J Zhejiang Agric Sci. (2024) 36:1279–89. Available online at: https://link.cnki.net/urlid/33.1151.S.20240517.2006.015

[41] Ding J Dai R Yang L He C Xu K Liu S . Inheritance and establishment of the gut microbiota in chickens. Front Microbiol. (2017) 8:1967. doi: 10.3389/fmicb.2017.01967 29067020 PMC5641346

[42] Stanley D Geier MS Denman SE Haring VR Crowley TM Hughes RJ . Identification of chicken intestinal microbiota correlated with the efficiency of energy extraction from feed. Vet Microbiol. (2013) 164:85–92. doi: 10.1016/j.vetmic.2013.01.018 23434185

[43] Liu H Zhao J Zhang W Nie C. Impacts of sodium butyrate on intestinal mucosal barrier and intestinal microbial community in a weaned piglet model. Front Microbiol. (2023) 14:1041885. doi: 10.3389/fmicb.2023.1041885 PMC9879053 36713180

[44] Cao J Guo Y Luo X Wu L Hu C Liu S . Interactions between enzyme preparations and trace element sources affect the growth performance and intestinal health of broilers. Anim Feed Sci Technol. (2023) 301:115726. doi: 10.1016/j.anifeedsci.2023.115726 PMC10651683 37922857

[45] Roberfroid M Gibson GR Hoyles L McCartney AL Rastall R Rowland I . Prebiotic effects: metabolic and health benefits. Br J Nutr. (2010) 104:S1–S63. doi: 10.1017/S0007114510003363, PMID: 20920376

[46] Gonçalves P Araujo JR Di Santo JP. Crosstalk between microbiota-derived short-chain fatty acids and the host mucosal immune system regulates intestinal homeostasis and inflammatory bowel disease. Inflamm Bowel Dis. (2018) 24:558–72. doi: 10.1093/ibd/izx029, PMID: 29462379

[47] Cui Y Han C Li S Geng Y Wei Y Shi W . High-throughput sequencing−based analysis of the intestinal microbiota of broiler chickens fed with compound small peptides of Chinese medicine. Poult Sci. (2021) 100:100897. doi: 10.1016/j.psj.2021.100897 33518313 PMC7936118

[48] Fujio-Vejar S Vasquez Y Morales P Magne F Vera-Wolf P Ugalde JA . The gut microbiota of healthy Chilean subjects reveals a high abundance of the phylum Verrucomicrobia. Front Microbiol. (2017) 8:1221. doi: 10.3389/fmicb.2017.01221, PMID: 28713349 PMC5491548

[49] Liu S Zhou W Deng X Jiang W Wang Y Zhan J . *Inonotus obliquus* polysaccharide are linear molecules that alter the abundance and composition of intestinal microbiota in Sprague Dawley rats. Front Nutr. (2023) 10:1231485. doi: 10.3389/fnut.2023.1231485, PMID: 37841402 PMC10568496

[50] Xiao Y Xiang Y Zhou W Chen J Li K Yang H. Microbial community mapping in the intestinal tract of broiler chickens. Poult Sci. (2017) 96:1387–93. doi: 10.3382/ps/pew372 28339527

[51] Wang X Li D Xu Y Ding X Liang S Xie L . Xylanase supplement enhances the growth performance of broilers by modulating serum metabolism, intestinal health, short-chain fatty acid composition, and microbiota. Animals. (2024) 14:1182. doi: 10.3390/ani14081182, PMID: 38672330 PMC11047501

[52] Liu T Ruan S Mo Q Zhao M Wang J Ye Z . Evaluation of the dynamic effects of dietary medium-chain monoglycerides on the performance, intestinal development and gut microbiota of broilers in large-scale production. Anim Nutr. (2023) 9:734–47. doi: 10.1016/j.aninu.2023.05.003 PMC10432913 37600838

[53] Yang T Chen S Qiu L Guo Q Wang Z Jiang Y . Effect of high dietary Iron on fat deposition and gut microbiota in chickens. Animals. (2024) 14:2254. doi: 10.3390/ani14152254, PMID: 39123780 PMC11310990

[54] Del Chierico F Abbatini F Russo A Quagliariello A Reddel S Capoccia D . Gut microbiota markers in obese adolescent and adult patients: age-dependent differential patterns. Front Microbiol. (2018) 9:1210. doi: 10.3389/fmicb.2018.01210, PMID: 29922272 PMC5996250

[55] Satam H Joshi K Mangrolia U Waghoo S Zaidi G Rawool S . Next-generation sequencing technology: current trends and advancements. Biology. (2023) 12:997. doi: 10.3390/biology12070997, PMID: 37508427 PMC10376292

[56] Bedford MR Apajalahti JH. The role of feed enzymes in maintaining poultry intestinal health. J Sci Food Agric. (2022) 102:1759–70. doi: 10.1002/jsfa.11670, PMID: 34802157 PMC9300167

[57] Dobi A Gasque P Guiraud P Selambarom J. Irinotecan (CPT-11), a canonical anticancer drug, can also modulate the antiviral and proinflammatory responses of primary human synovial fibroblasts. Cells. (2021) 10:1431. doi: 10.3390/cells10061431 34201243 PMC8230279

[58] Jung JH Lee D Ko HM Jang HJ. Inhibition of CNOT2 induces apoptosis via MID1IP1 in colorectal cancer cells by activating p53. Biomolecules. (2021) 11:1492. doi: 10.3390/biom11101492, PMID: 34680125 PMC8533695

[59] Maya-Sandino A Lozada-Soto KM Rajagopal N Garcia-Hernandez V Luissint AC Brazil JC . Claudin-23 reshapes epithelial tight junction architecture to regulate barrier function. Nat Commun. (2023) 14:6214. doi: 10.1038/s41467-023-41999-9 37798277 PMC10556055

[60] Sharygin D Koniaris LG Wells C Zimmers TA Hamidi T. Role of CD14 in human disease. Immunology. (2023) 169:260–70. doi: 10.1111/imm.13634, PMID: 36840585 PMC10591340

[61] Zuo W Rostami MR Shenoy SA LeBlanc MG Salit J Strulovici-Barel Y . Cell-specific expression of lung disease risk-related genes in the human small airway epithelium. Respir Res. (2020) 21:200–11. doi: 10.1186/s12931-020-01442-9, PMID: 32727470 PMC7389881

[62] Liu Z Liu LZ Sun CJ Sun C Yan Y Li G . Genome-wide association analysis of age-dependent egg weights in chickens. Front Genet. (2018) 9:128. doi: 10.3389/fgene.2018.00128, PMID: 29755503 PMC5932955

[63] Fontana BD Norton WHJ Parker MO. Modeling ADHD-like phenotypes in Zebrafish In: New discoveries in the behavioral neuroscience of attention-deficit hyperactivity disorder. Cham: Springer International Publishing (2022). 395–414. 10.1007/7854_2022_343 35507286

[64] Wang Q Chu F Zhang X Hu H Lu L Wang F . Infectious bursal disease virus replication is inhibited by avain T cell chemoattractant chemokine CCL19. Front Microbiol. (2022) 13:912908. doi: 10.3389/fmicb.2022.912908, PMID: 35935208 PMC9355407

[65] Hong Y Cheng Y Guan L Zhou Z Li X Shi D . *Bacillus amyloliquefaciens* TL downregulates the ileal expression of genes involved in immune responses in broiler chickens to improve growth performance. Microorganisms. (2021) 9:382. doi: 10.3390/microorganisms9020382, PMID: 33668643 PMC7918048

[66] Azli B Ravi S Hair-Bejo M Omar AR Ideris A Mat Isa N. Functional prediction of de novo unigenes from chicken transcriptomic data following infectious bursal disease virus at 3-days post infection. BMC Genomics. (2021) 22:461. doi: 10.1186/s12864-021-07690-3 34147086 PMC8214787

[67] Park YB Lim C Lim B Kim JM. Long noncoding RNA network for lncRNA–mRNA interactions throughout swine estrous cycle reveals developmental and hormonal regulations in reproductive tissues. J Anim Sci Technol. (2024) 66:1109–24. doi: 10.5187/jast.2023.e137 39691614 PMC11647408

[68] Mitra T Bramberger B Bilic I Hess M Liebhart D. Vaccination against the protozoan parasite *Histomonas meleagridis* primes the activation of toll-like receptors in turkeys and chickens determined by a set of newly developed multiplex RT–qPCRs. Vaccine. (2021) 9:960. doi: 10.3390/vaccines9090960, PMID: 34579197 PMC8472887

[69] Chuwatthanakhajorn S Chang CS Ganapathy K Tang PC Chen CF. Comparison of immune-related gene expression in two chicken breeds following infectious bronchitis virus vaccination. Animals. (2023) 13:1642. doi: 10.3390/ani13101642, PMID: 37238072 PMC10215283

[70] Xue L Chen S Xue S Xue LX Chen SF Xue SX . P2RY2 alleviates cerebral ischemia–reperfusion injury by inhibiting YAP phosphorylation and reducing mitochondrial fission. Neuroscience. (2022) 480:155–66. doi: 10.1016/j.neuroscience.2021.11.013, PMID: 34780922

[71] Guo Y Su A Tian H Zhai M Li W Tian Y . Transcriptomic analysis of spleen revealed mechanism of dexamethasone-induced immune suppression in chicks. Genes. (2020) 11:513. doi: 10.3390/genes11050513, PMID: 32384708 PMC7288455

[72] Prentice KJ Saksi J Hotamisligil GS. Adipokine FABP4 integrates energy stores and counterregulatory metabolic responses. J Lipid Res. (2019) 60:734–40. doi: 10.1194/jlr.091185 30705117 PMC6446704

[73] Hasumi Y Baba M Hasumi H Huang Y Lang M Reindorf R . Folliculin (Flcn) inactivation leads to murine cardiac hypertrophy through mTORC1 deregulation. Hum Mol Genet. (2014) 23:5706–19. doi: 10.1093/hmg/ddu286, PMID: 24908670 PMC4189904

[74] Yang Q Zhang P Han L Shi P Zhao Z Cui D . The mitochondria-related genes PDK2, CHDH, and ALDH5A1 serve as diagnostic signatures and are correlated with immune cell infiltration in ulcerative colitis. Aging. (2024) 16:3803–21. doi: 10.18632/aging.205561 38376420 PMC10929806

[75] Fernandes ES Fernandes MA Keeble JE. The functions of TRPA1 and TRPV1: moving away from sensory nerves. Br J Pharmacol. (2012) 166:510–21. doi: 10.1111/j.1476-5381.2012.01851 22233379 PMC3417484

[76] Armstrong E Iriarte A Nicolini P de Los Santos J Ithurralde J Bielli A . Comparison of transcriptomic landscapes of different lamb muscles using RNA-Seq. PLoS One. (2018) 13:e0200732. doi: 10.1371/journal.pone.0200732, PMID: 30040835 PMC6057623

[77] Du Y Cao C Liu Y Zi X He Y Shi H . Polymorphism, genetic effect, and association with egg-laying performance of Chahua chickens matrix metalloproteinases 13 promoter. Genes. (2023) 14:1352. doi: 10.3390/genes14071352, PMID: 37510257 PMC10379211

[78] Malhotra A Ziegler A Shu L Perrier R Amlie-Wolf L Wohler E . De novo missense variants in *LMBRD2* are associated with developmental and motor delays, brain structure abnormalities and dysmorphic features. J Med Genet. (2020) 58:712–6. doi: 10.1136/jmedgenet-2020-107137, PMID: 32820033 PMC11431178

[79] Monson MS Coulombe RA Reed KM. Aflatoxicosis: lessons from toxicity and responses to aflatoxin B1 in poultry. Agriculture. (2015) 5:742–77. doi: 10.3390/agriculture5030742

[80] Biţă A Scorei IR Ciocîlteu MV Nicolaescu OE Pîrvu AS Bejenaru LE . Nicotinamide riboside, a promising vitamin B3 derivative for healthy aging and longevity: current research and perspectives. Molecules. (2023) 28:6078. doi: 10.3390/molecules28166078, PMID: 37630330 PMC10459282

[81] Lindholm C Batakis P Altimiras J Lees J. Intermittent fasting induces chronic changes in the hepatic gene expression of red jungle fowl (*Gallus gallus*). BMC Genomics. (2022) 23:304. doi: 10.1186/s12864-022-08533-5, PMID: 35421924 PMC9009039

[82] Jiang S Zou X Mao M Zhang M Tu W Jin M. Low ca diet leads to increased ca retention by changing the gut flora and ileal pH value in laying hens. Anim Nutr. (2023) 9:534–45. doi: 10.1016/j.aninu.2023.02.006 PMC10164782 37168452

