# Supplementary figures and images for: High-value valorization of Periplaneta americana residue: intestinal health regulation in pre-bred hens and circular economy applications
Source: Front Vet Sci. 2025 Dec 11;12:1700996. doi: 10.3389/fvets.2025.1700996 (PMC12739957; doi:10.3389/fvets.2025.1700996)

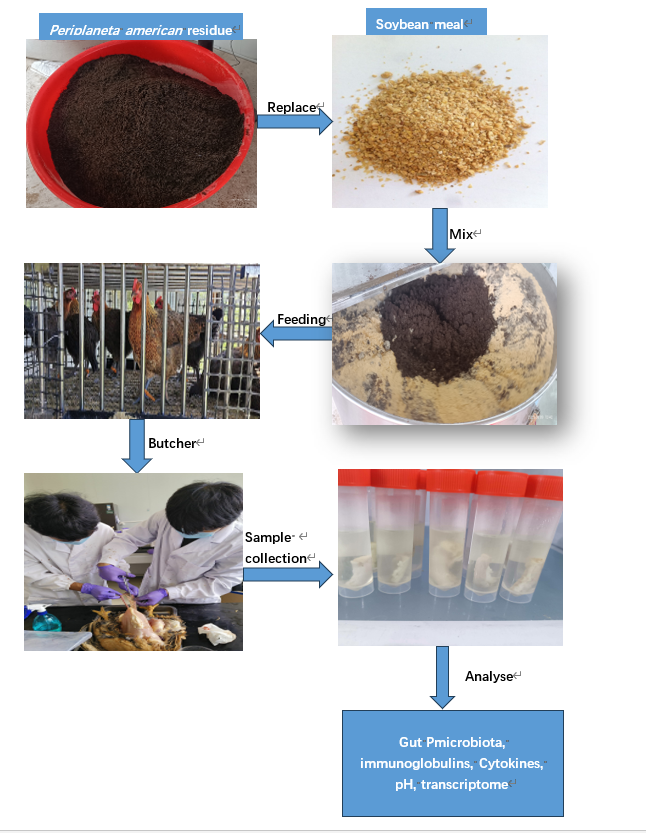

Supplement: Supplementary file 1 [file Image_1.tiff]

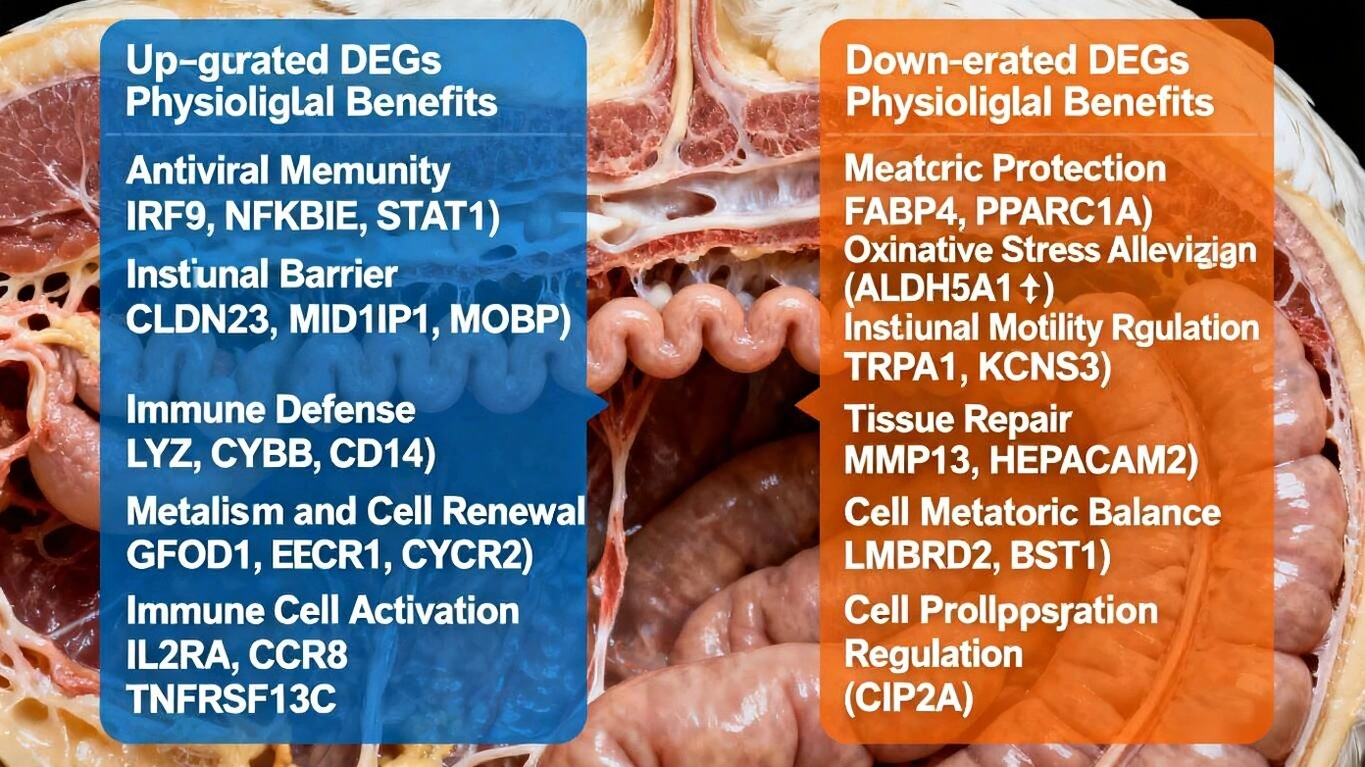

Supplement: Supplementary file 2 [file Image_2.tiff]
